# SUMO-mediated recruitment allows timely function of the Yen1 nuclease in mitotic cells

**DOI:** 10.1371/journal.pgen.1009860

**Published:** 2022-03-25

**Authors:** Hugo Dorison, Ibtissam Talhaoui, Gerard Mazón

**Affiliations:** 1 Université Paris-Saclay–UMR9019 CNRS–Gustave Roussy, Villejuif, France; 2 Inserm–Institut National de la Santé et de la Recherche Médicale, Paris, France; Duke University, UNITED STATES

## Abstract

The post-translational modification of DNA damage response proteins with SUMO is an important mechanism to orchestrate a timely and orderly recruitment of repair factors to damage sites. After DNA replication stress and double-strand break formation, a number of repair factors are SUMOylated and interact with other SUMOylated factors, including the Yen1 nuclease. Yen1 plays a critical role in ensuring genome stability and unperturbed chromosome segregation by removing covalently linked DNA intermediates between sister chromatids that are formed by homologous recombination. Here we show how this important role of Yen1 depends on interactions mediated by non-covalent binding to SUMOylated partners. Mutations in the motifs that allow SUMO-mediated recruitment of Yen1 impair its ability to resolve DNA intermediates and result in chromosome mis-segregation and increased genome instability.

## Author summary

The modification of proteins with the small SUMO peptide enables the appropriate localization and correct organization of multiple factors during the repair of DNA damages, including double-strand breaks that could sever the DNA molecule that contains the genetic information of cells. In this work, we describe how the Yen1 protein, a DNA repair factor involved in the last steps of the key DNA repair mechanism called homologous recombination, is controlled by SUMO modification and by its ability to contact other SUMO-modified proteins through specific SUMO interaction motifs (SIMs) in its C-terminal domain. Impairing the SUMO interactions mediated by Yen1’s SIMs results in a deficient function of this factor during the resolution of joint DNA molecules that would otherwise prevent the chromosomes to fully separate and segregate to the two daughter cells during each cell division. In living cells, this defect in Yen1 functions related to the absence of interaction with SUMO results in defective protein localization, increased sensitivity to DNA damaging agents, and increased chromosome segregation problems. This SUMO-mediated control mechanism of the Yen1 nuclease is thus important to preserve genome integrity.

## Introduction

Genome integrity is constantly threatened by multiple challenges, either from endogenous or exogenous sources of DNA damage. Through evolution, cells have acquired multiple DNA repair pathways to ensure genome stability, homologous recombination (HR) being one of the most critical pathways to counter the deleterious DNA double-strand breaks (DSBs) and other lesions arising during replication. As the HR pathway operates, different DNA substrates and intermediates form that physically inter-connect distinct DNA molecules, creating a joint-molecule (JM) intermediate. These intermediates are a threat to the successful segregation of chromosomes and are to be dismantled during mitosis by different specialized proteins acting in concert to prevent segregation defects and genome rearrangements. In yeast, the dissolution pathway mediated by the Sgs1-Top3-Rmi1 (STR) complex ensures the disentanglement and release of double Holliday junctions (dHJs), and two other helicases, Mph1 and Srs2, act early on the pathway, preferentially over D-loop intermediates, to reduce the number of JM intermediates and ensure the completion of the recombinational repair without crossing over between the involved DNA templates. Opposed to these non-crossover (NCO) pathways, the nucleolytic processing of these JM intermediates can result in reciprocal crossovers (COs), with the risk of genome rearrangements and loss of heterozygosity (LOH) events [1,2].

Given the risk for genome stability of a nucleolytic processing of HR intermediates, the different actors able to cleave these intermediates are strictly controlled and used as an option of last resort to resolve DNA substrates not previously dismantled by the action of helicases [3,4]. Two major nucleases are involved in the nucleolytic processing of recombination intermediates in the yeast model, Mus81-Mms4 and Yen1 [5]. The Mus81-Mms4 nuclease plays different roles at replication forks, and is gradually hyper-activated by Cdc5- and Cdc28/Cdk1-dependent phosphorylation of Mms4 to peak its activity in late G2/M [6–8], where it associates with the Slx4-Dpb11 scaffold [9]. Thanks to broad substrate recognition, Mus81-Mms4 can cleave 3’-flap containing DNA substrates and HJs, preferentially when they are still nicked or not completely ligated [10]. The hyper-activation of Mus81-Mms4 in late G2 and its broad substrate specificity enable Mus81-Mms4 to target captured D-loops and early HJ intermediates that are not completely converted into closed dHJ and thus remain inaccessible to processing by the STR complex [3]. As mitosis progresses, the Cdc14 phosphatase will trigger the reversal of the inhibitory Cdc28-mediated phosphorylation of Yen1, in turn allowing its nuclear localization and its proper substrate recognition [11,12]. This late activation of Yen1 at the anaphase entry ensures that all remaining recombination intermediates, especially those that escaped dissolution by STR or cleavage by Mus81-Mms4, are resolved before mitotic exit [11,12]. To ensure the clearing of Yen1 from the nucleus in the subsequent S-phase, and prevent off-targeted activity directed to 5’-flap containing DNA intermediates, Yen1 is additionally controlled by a SUMO-targeted degradation mediated by the Slx5-Slx8 ubiquitin ligase, further limiting the potential of crossover formation [13].

Protein covalent modification with the small ubiquitin-like modifier (SUMO) [14] is an important mechanism to fine tune DNA transactions during the DNA damage and repair responses [15–18]. In *Saccharomyces cerevisiae*, SUMOylation occurs in a multi-step reaction involving the E1 Aos1-Uba2 activating enzyme dimer, the E2 conjugating enzyme Ubc9, and three possible E3 ligases (Siz1, Siz2 and Mms21), with some redundancy of Siz1 and Siz2 for their substrates [19–21]. Several players of the HR pathway, besides the Yen1 nuclease, are also found among the SUMOylated DNA repair targets, including Rad52, PCNA, RPA and Sgs1 [17,22–26]. SUMOylation can influence biological processes in different ways. Proteins can be mono-SUMOylated, multi-SUMOylated or poly-SUMOylated, and the modification will re-design the protein surfaces, allowing changes in protein activity, or in the way it can interact with other proteins. One of the best-described effects of protein SUMOylation is the enabling of interaction with other protein partners in a bait-to-prey fashion, using SUMO as the moiety that is recognized by a specific domain in the partner protein, called a SUMO interacting motif (SIM). These motifs are found throughout species and, according to their amino acid composition, are classified in several families of consensus sequences [27]. Most SIMs can be defined as a core stretch of four amino acids with a majority of hydrophobic residues (typically rich in V/I/L). This hydrophobic core fits into the hydrophobic groove on the SUMO surface and is often flanked by a stretch of 3–4 acidic or polar residues in the SIM sequence that interact with basic residues on the surface of SUMO [28–31]. SIM types showing the flanking stretch of acidic residues present thus a similar architecture to that of ubiquitin interacting motifs (UIM) that also show a key stretch of polar residues flanking the core [32,33]. SIM motifs can interact with mono- or poly-SUMOylated proteins and can be usually present in tandem dispositions, probably helping interaction with multiple SUMOylated lysines or with a poly-SUMOylated lysine in the interacting protein [28,34,35]. Interactions by SUMO-SIM partnerships are extremely labile and can be easily induced and curbed down by altering the SUMOylation status of the involved proteins. This flexibility allows a quick building of protein complexes in response to changing stress conditions in the cell [17,35]. The formation of these protein complexes by SUMO-mediated recruitment *via* SIMs is often associated with the actual SUMOylation of the two involved proteins [36,37]. Then, it was not surprising to identify in Yen1, which is SUMOylated [13], several putative SIMs. In the present study, we define two functional SIMs in the Yen1 C-terminal region that play important roles in the protein sub-nuclear localization and its function alleviating the persistence of chromosome-segregation challenging JMs throughout the end of mitosis.

## Results

### Yen1 contains two functional SUMO interacting motifs (SIM) in the C-terminal region

**Fig 1:**
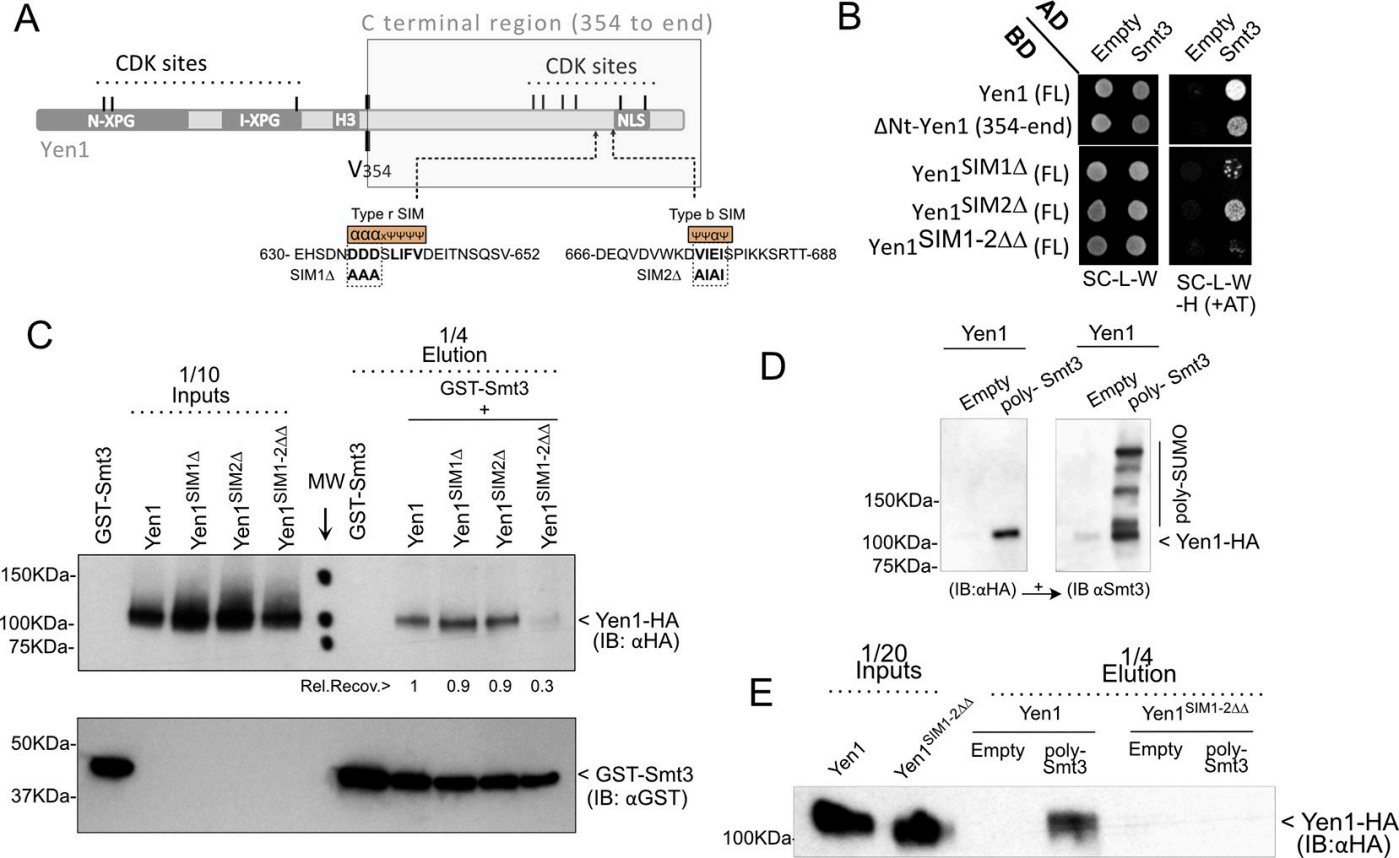
Yen1 contains two SUMO Interacting Motifs (SIMs) in its C-terminal domain. **(A)** Diagram showing the conserved domains of Yen1 and the positions of the regulatory Cdk1-phosphorylation sites. Amino acid 354 shows the cut-off point for truncated forms of Yen1 in Two-Hybrid assays. The two identified candidate SIMs are shown near the Nuclear localization sequence (NLS). **(B)** A Yeast two-hybrid assay was performed with strains carrying the indicated Activator Domain (AD) and DNA Binding Domain (BD) fusions to test interaction between Yen1 and Smt3 (SUMO), and the Yen1 critical domains for such interaction. Mutations D635A D636A D637A for SIM1Δ and V675A E677A for SIM2Δ were introduced to test for the putative SIMs. SIM1-2ΔΔ is used for the combined mutations. Strains were grown on selective media lacking leucine and tryptophan and spotted in selective media also lacking histidine to reveal interaction of the proteins fused to the AD and BD domains. Non-specific interactions were minimized by the addition of 3-aminotriazole (AT). **(C)** Purified GST-Smt3 was bound to a Glutathione resin, and either purified wild-type or the SIM mutant Yen1 proteins were then loaded to the resin. The retained fractions of Yen1 were eluted after several washes and detected by immuno-blotting. **(D)** SUMO-retention assay using *in vitro* generated poly-Smt3 immobilized into a Cobalt HisPur Superflow agarose matrix. Purified Yen1 was added to the pre-bound Smt3 and after a binding-time and washing, columns were eluted in denaturing conditions and the eluates inspected by western blot for the presence of Yen1 (anti-HA, left panel) and the pre-bound Smt3 chains (right panel). **(E)** Immuno-blotting of the inputs and eluates of the retention assay (as in D) comparing the ability of wild-type or SIM-mutant Yen1 to bind poly-Smt3.

As stressed before, SUMOylated proteins are able to interact transiently with a variable strength with other proteins containing SIMs. These motifs consist of a stretch of amino acids with a core of aliphatic residues, often flanked by three or more amino acids with a negative charge or susceptible to becoming phosphorylated [27]. We inspected the Yen1 sequence through available algorithms [27] to detect SIM motifs, and we identified several interesting hits in the primary sequence of Yen1 (Fig 1A). To validate such motifs in Yen1, we performed a yeast two-hybrid analysis with the SUMO encoding gene Smt3 as bait and either wild-type full-length or truncated versions of Yen1 as prey (Fig 1B). The ability to interact with SUMO in the yeast two-hybrid assay was only retained by the C-terminal part of Yen1 (amino acids 354 to 759) and mutations in two putative SIMs present in this half of the protein completely abolished the interaction. A mutation in the first SIM, a type r motif [27] with the hydrophobic core at amino acids 636 to 642 next to its defining stretch of acidic residues that is thought to dictate the orientation of the interaction of SUMO to this type of SIMs [34,38], was indeed sufficient to almost completely impair the interaction with Smt3 in the yeast two-hybrid assay (Fig 1B). Interestingly, this motif is highly conserved across Yen1 in other fungi and is very reminiscent of SIMs found in the Slx5, Rad18 and Elg1 proteins [36,39,40] (S1 Fig). Mutation in a secondary SIM2 (675 to 678) further decreased the interaction in the yeast two-hybrid assay when combined with the mutation in SIM1 (Fig 1B).

While mutation in the consensus SIM sites abolishes the interaction in the yeast two-hybrid assay, a previous result showing interaction of Yen1 with Smt3 in this assay was suggested to reflect a covalent modification of Yen1 [41]. The absence of interaction that we detected in this test might then reflect the loss of direct SUMOylation of Yen1, related to the absence of non-covalent interaction of Yen1 with SUMO through its SIMs. A SIM-mediated interaction would be difficult to confirm in this test if it requires polymerization of Smt3 or a SUMO covalent modification of a bridging partner, ultimately resulting in the growth read-out of the test. To better confirm the nature of the interaction lost in our mutants and thus validate the SIM sites, we used a pull-down approach [36]. GST-Smt3 was over-expressed and purified from bacteria, and bound to a glutathione resin. Purified Yen1 or its mutant SIM variants were then allowed to bind to the pre-bound GST-Smt3, and after several washes, the column content was eluted in denaturing conditions and inspected by western blotting (Fig 1C). Yen1 was detected in the eluates thus confirming its ability to interact non-covalently with Smt3. Much less Yen1 was retained (30% compared to wild-type) when bearing mutations in both of its SIMs, while inactivation of only one of the two motifs did not significantly alter retention, suggesting that, at least *in vitro*, one single SIM at the Yen1 C-terminal region confers its ability to bind Smt3 monomers (Fig 1C). The presence of two SIMs may help stabilize interactions with bi-sumoylated or poly-sumolylated proteins. We decided to further evaluate whether Yen1 can be captured by poly-Smt3 using a Cobalt HisPur Superflow affinity column that was previously loaded with poly-SUMO chains generated *in vitro* in the presence of Aos1-Uba2, Ubc9 and purified 6xHis-Smt3. This poly-Smt3 coated matrix was used to test retention of Yen1-1xHA (Fig 1D). The recovery of the protein mutated in both SIMs (Yen1^SIM1-2ΔΔ^) was greatly decreased (Fig 1E), thus confirming that Yen1 binds non-covalently to poly-SUMO chains, a binding that depends on the presence of the two identified Yen1 SIMs.

### Strains carrying SIM-defective variants of Yen1 display increased sensitivity to DNA damage

**Fig 2:**
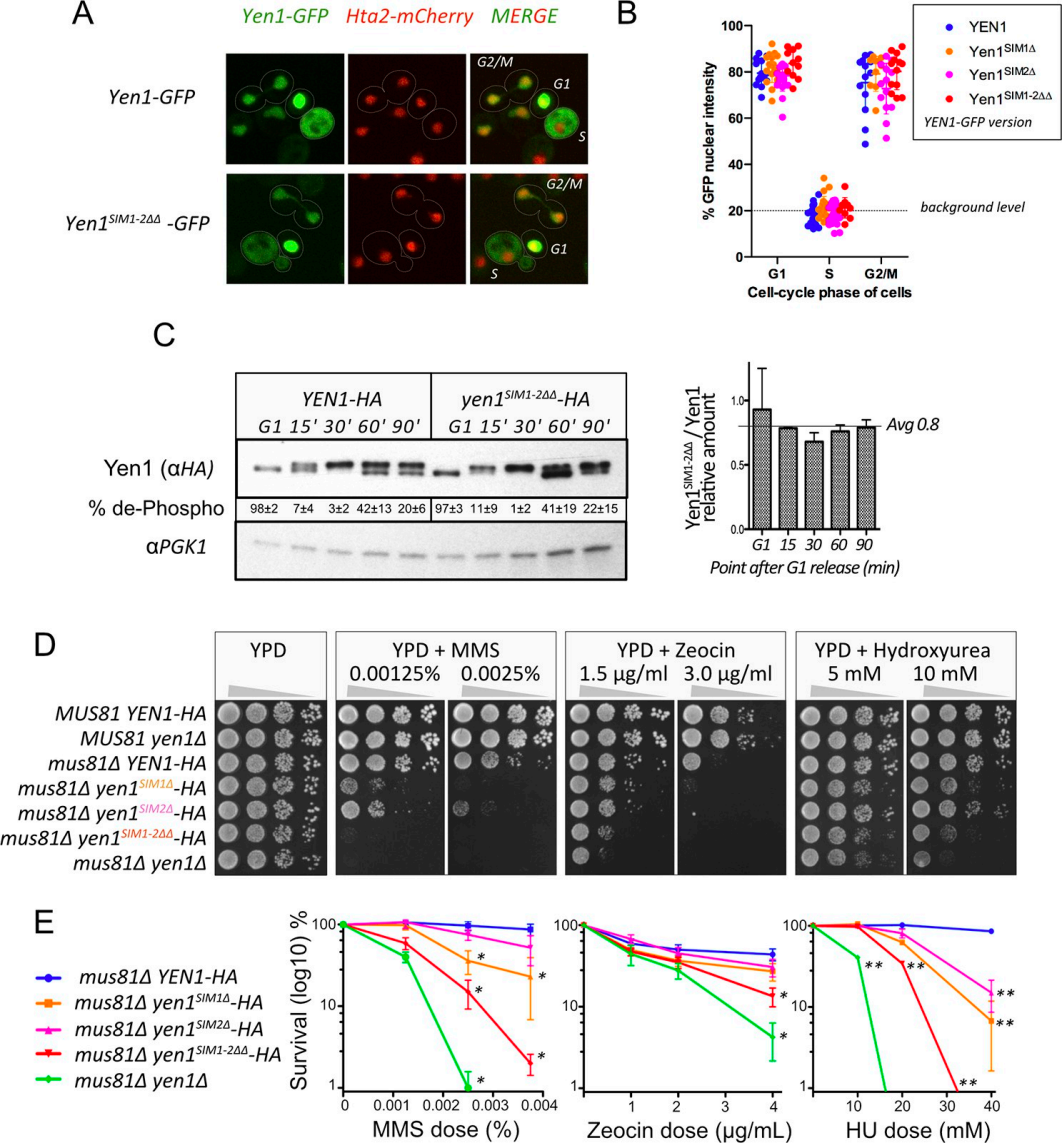
Mutation in Yen1 SIMs has no impact to its CDK1 regulation and nuclear shuttling but sensitizes cells to DNA damage. **(A)** Cells carrying an endogenous histone Hta2-mCherry marker and chromosomally–HA tagged versions of Yen1 wild-type and the different SIM mutants were transformed with a plasmid carrying an equivalent version of Yen1 fused with GFP at its C-terminal region. Cells were grown on selective media and observed using a spinning-disk microscope after a brief induction with galactose. Shuttling of the protein from cytoplasm to the nucleus can be observed in representative fields displaying cells with nuclear excluded Yen1 (S-phase and early G2-M) and nuclear localized Yen1 (anaphase to G1) for the indicated GFP-tagged proteins. **(B)** Quantification of the relative amount of GFP signal detected into the nucleus over the overall signal for the indicated strains in cells classified by their cell-cycle status **(C)** Strains with a chromosomally inserted copy of–HA tagged wild-type Yen1 or its double SIM mutant (Yen1^SIM1-2ΔΔ^) were synchronized with alpha factor and released into fresh medium to monitor the modification of the protein through the cell cycle by immunoblot (left). Both unmodified and phosphorylated Yen1 are indicated. Average levels of endogenous Yen1 were normalized with PGK1 protein in triplicate experiments (right). **(D)** Sensitivity to different DNA damaging agents and drugs was determined by spotting serial dilutions of strains carrying different Yen1 mutants in its SIM in a *MUS81* deleted background for the indicated media. **(E)** Survival curves to the agents tested in (C) were established by counting colony forming units of the different strains after plating in YPD containing the indicated doses of drugs in replicate trials. Survival was normalized per trial with its respective control YPD counts and the average survival is plotted in the graphs (+/- SEM). Significance was estimated by the student T-test at P<0.05 (*) and P<0.01 (**), see S3 Table. Additional data related to this figure are found in S2 Fig.

We next aimed to understand the effect of the mutations in the SIM motifs on the normal regulation of Yen1 by Cdk1/Cdc14 and its timely localization to the nucleus. Given the proximity of Yen1’s SIMs to its nuclear localization signal (NLS), we monitored a C-terminal GFP fusion of the Yen1 mutants to see if any gross nuclear shuttling defects occurred (Fig 2). Both single and double SIM mutants presented nuclear exclusion in S-phase, as the wild-type, and were nuclear in late mitosis and G1. There were no changes in the relative distribution of GFP intensity detected in the nucleus or in the cytoplasm at the different cell cycle phases between the different Yen1 variants (Figs 2A, 2B and S2). We also analyzed the pattern of cyclic phosphorylation by synchronizing cells in G1 and analyzing the mobility of Yen1 at different time points after its release (Figs 2C and S2A). All the mutants displayed a normal cycle of phosphorylation in S-phase followed by gradual de-phosphorylation with only slight variations in the total amount of the protein across all cell-cycle phases. Next, we wondered whether the presence of an endogenous copy of the SIM mutants would compromise the ability of Yen1 to back up for the functions of Mus81-Mms4 [5]. The mutants were introduced into a *mus81*Δ background and the resultant strains were tested for their sensitivity to an array of DNA damaging agents (Fig 2D and 2E). Cells with a double deletion *mus81Δ yen1Δ* are extremely sensitive to MMS at low doses, and they also present a moderate sensitivity to the radiomimetic drug Zeocin and to the replication stalling drug Hydroxyurea (HU) [13]. Mutations in the first SIM or simultaneously in both SIMs significantly increased the sensitivity of a *mus81*Δ strain to MMS, while increased sensitivity to MMS after mutation in the second SIM alone was not significant (Fig 2D, 2E and S3 Table). Mutation in both SIMs was necessary to see a moderate increase in the sensitivity of cells to Zeocin (Fig 2D and 2E). Mutation in both SIMs was also necessary to sensitize cells at 20 mM HU and, while individual SIM mutants sensitized cells to 40 mM HU, at such dose survival was already compromised by a two log difference in cells bearing both SIM mutations or lacking *YEN1* (Fig 2E). Our results point to a preeminent role of SIM1, conferring stronger phenotypes than the SIM2 single mutant. We wondered whether the substitution of the central hydrophobic core would have stronger effects than mutating the acidic stretch of SIM1, but we obtained the same results with both sets of mutations (S3 Fig). Yen1 has also been described to be essential in cells with a deletion in the *DNA2* helicase-nuclease in the presence of its suppressor *pif1*Δ [42–45]. Similarly, cells carrying the *dna2-2* helicase-deficient allele rely on the activity of *YEN1* to process DNA intermediates in this background [42]. Similar to what was already observed in *dna2-2* cells in the W303 background [42], we found that a *dna2-2* mutation is partially unviable with *yen1Δ* (S4 Fig). The *dna2*-2 cells, while viable, present a strong heterogeneity of phenotypes likely associated with spontaneous accumulation of suppressors, as described [42] (S4 Fig). Nonetheless, the introduction of the most severe of the SIM alleles, carrying simultaneous mutations in both SIMs, was viable in a *dna2-2* background, suggesting a minor role of non-covalent SUMO binding for the Yen1’s functions required in a *dna2-2* context. In agreement with the observation that *dna2Δ pif1Δ* strains do not significantly accumulate more SUMOylated Yen1 compared to the wild-type strain [13], we found that in a *dna2Δ pif1Δ* background the SIM defective allele does not increase the already strong sensitivity of cells to DNA damaging agents like MMS or HU (S4 Fig).

### Mutation of the SIMs induces a SUMO-less Yen1 phenotype *in vivo*

**Fig 3:**
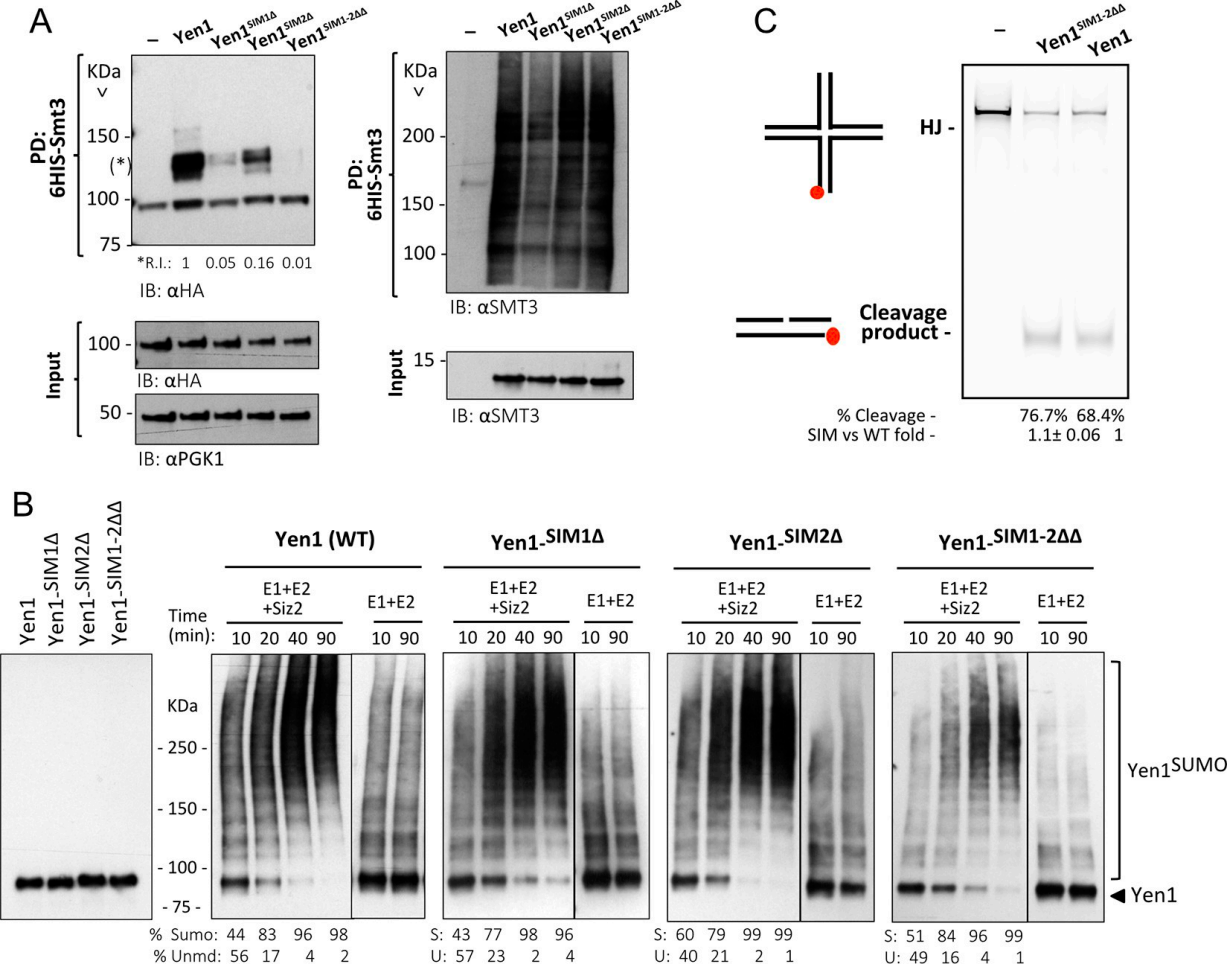
Mutation in Yen1 SIMs does not alter its activity or its SUMOylation *in vitro* but prevents SUMOylation *in vivo*. **(A)** Strains carrying endogenous copies of–HA tagged wild type Yen1, Yen1^SIM1Δ^, Yen1^SIM2Δ^ and Yen1^SIM1-2ΔΔ^ mutants, with or without (-) the plasmid pCUP-6xHIS-Smt3, were grown in the presence of MMS 0.3%. Cells were lysed and lysates subjected to a denaturing Ni-NTA pull-down followed by immunoblot analysis. Yen1 was detected by anti-HA. Membranes were subsequently probed with anti-Smt3. Prior to Ni-NTA pull-down, input samples were taken from the lysates and were analyzed by immunoblotting for the levels of Smt3 induction (Anti-Smt3) and relative protein amounts (Anti-PGK1, Anti-HA) of each lysate (input panels). **(B)** Purified Yen1-HA and Yen1 SIM-mutant variants were subjected to an *in vitro* SUMOylation reaction containing Siz2, Aos1-Uba2, Ubc9 and Smt3-3KR and ATP and subjected to Tris-Acetate PAGE for comparison of their SUMOylation patterns after immunoblotting with anti-HA. The reaction without the ligase Siz2 is shown at the right of each panel. Quantification of the relative % of SUMOylation forms (R.I.) and unmodified forms is shown at the bottom and more in detail in S4 Fig **(C)** Activity of Immuno-precipitated Yen1 was tested in a cleavage reaction using synthetic Holliday junctions (HJ) made with oligonucleotides and labeled with Cy5. The DNA products were run in non-denaturing PAGE and revealed by the fluorescence of the Cy5 labeled oligonucleotide.

In other SUMOylated DNA repair proteins containing functional SIMs, the mutation of these motifs has an impact on the ability of the protein to be directly SUMOylated [36,37]. The results in the yeast two-hybrid experiments suggested such an effect for Yen1 (Fig 1B), which we had previously characterized to be SUMOylated in a Siz1/Siz2-dependent manner [13]. To further confirm the absence of covalent SUMOylation after impairment of Yen1’s SIMs we compared the SUMOylation levels of the wild-type and the SIM-defective Yen1 mutants by performing denaturing pull-downs of His-tagged Smt3 (Fig 3A). Yen1 SUMOylation peaks when cells are exposed to high MMS doses [13] and we reproduced Yen1 SUMOylation in these conditions for the wild-type protein (Fig 3A). Nonetheless, the fraction of SUMOylated Yen1 in the mutant in either the first SIM motif or the double mutant in the two SIM motifs was greatly reduced in conditions with similar input levels to 5% and 1% of the wild-type levels respectively (Fig 3A). Mutation of SIM2 had a milder, although significant, effect reducing the recovery of SUMOylated forms to ≈ 20% of the forms recovered in the wild-type (Fig 3A). The gradual effects detected for individual or combined mutations of both SIMs points to a concerted action of both motifs to promote Yen1 SUMOylation by allowing Yen1 non-covalent binding to Smt3. Similar to the observed sensitivities to DNA damaging agents, the substitution of either the acidic stretch or the hydrophobic core of SIM1 generated the same results (S3C Fig). Despite their sensitivity to very low doses of MMS, cells carrying *dna2-2* did not spontaneously increase the yield of SUMOylated forms of Yen1 (S4D Fig). In these conditions reflecting SUMOylation in response to spontaneous DNA damages, the Yen1 protein containing mutations in both SIMs showed a reduced yield of SUMOylation in either a wild-type or a *dna2-2* background (S4D Fig).

The mutations introduced to inactivate the SIM sites do not contain any lysine substitutions, and SIM1 is not directly flanked by lysines in their immediate vicinity. To further confirm that the lack of SUMOylation was not due to inadvertent absence or inaccessibility of SUMOylation-target lysines in Yen1, we decided to test the mutant proteins in an *in vitro* SUMOylation reaction. After enzymatic reaction in the presence of Yen1 or its SIM mutants, Aos1-Uba2 and the conjugating enzyme Ubc9, a normal SUMOylation pattern was detected with the same ladder of bands with increasing sizes for all Yen1 variants (Fig 3B). We also performed a complete SUMO-ligation reaction containing Siz2 as E3, increasing then the yield of the reaction. Comparing the ligation reactions over time, we could not detect significant differences in the amount or timing of accumulation of the SUMOylated forms (Figs 3B and S5) that in all the Yen1 variants achieved complete SUMOylation of these substrates at similar time points. We conclude that the presence of the SIM mutations does not preclude modification of any of the Yen1’s lysines targeted by the SUMOylation machinery.

The C-terminal domain of Yen1 containing the two SIMs is dispensable for complete nuclease activity [46]. Nonetheless, we verified that the mutation of both SIMs does not impair Yen1’s nuclease activity *in vitro* by using a synthetic Holliday junction [47] as a substrate. Immuno-precipitated Yen1 was added to cleavage reactions, and we compared the yield of HJ cutting for either the wild-type Yen1 and the SIM defective mutant Yen1^SIM1-2ΔΔ^. The nuclease activity was indistinguishable for both the wild-type and the mutant, which were able to linearize the HJ substrate at similar rates (Fig 3C). Alteration of the SIM motifs at the C-terminal part of the protein thus seems not to alter the cutting efficiency of Yen1, whose nuclease and conserved XPG domains are present at the N-terminal part of the protein (Fig 1A). Nonetheless, the test did not take into account the disposition of the junction in a chromatin context in the cell that could influence the ability to cut HJ *in vivo*.

### Localization to spontaneous and induced sites of activity is impaired by inactivation of the Yen1’s SIMs

**Fig 4:**
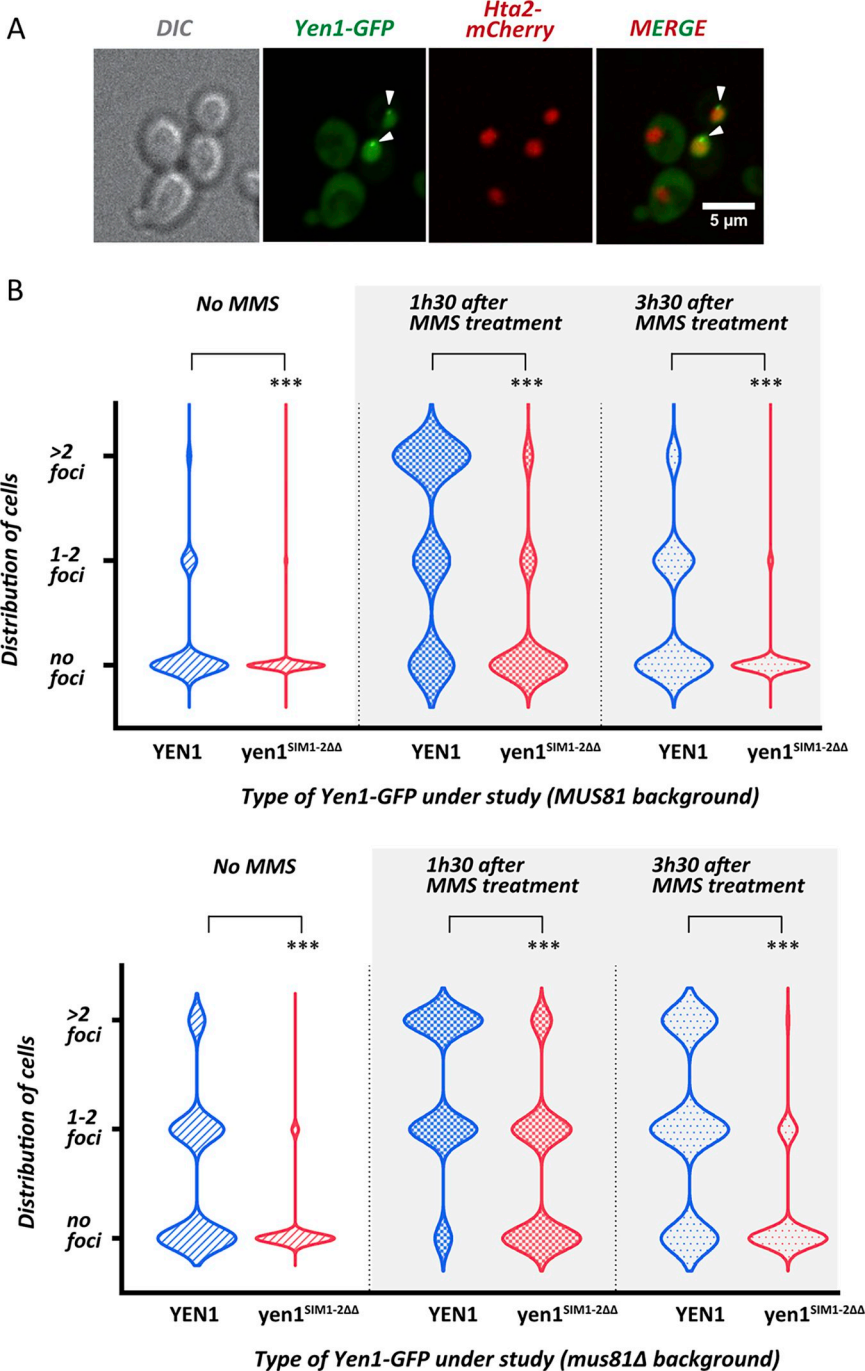
Mutation in the SIMs of Yen1 prevents foci accumulation in G2/M. **(A)** Cells with an endogenous copy of Hta2-mCherry and YEN1-HA expressing Yen1-GFP from an inducible vector were observed under a spinning-disk microscope after a brief induction with galactose. The white triangles denote the presence of Yen1-GFP foci. **(B)** Chromosomally tagged Yen1-HA wild-type and SIM mutant in the indicated genetic backgrounds and carrying its corresponding Yen1-GFP expressing plasmid (blue graphs for WT copy, red for SIM mutant) were observed under the microscope after a brief induction. Cells from the indicated conditions were classified according to its cell cycle phase and the presence or absence of Yen1 foci. Violin Plots display the distribution of G2/M cells showing no foci, 1–2 foci or more than 2 foci for each strain. Counting was performed for over 400 distinct G2/M cells for each strain over several independent trials. Asterisks represent statistical significance in a χ2 test p<0.0001 (***), see S4 and S5 Tables for details.

SUMOylation and interaction with SIMs have been proposed as a way to enforce a cascade of interactions to foster recruitment of factors to specific subcellular locations [35]. We decided to determine if the impairment of Yen1 SIMs was somehow altering the normal behavior of Yen1 by studying its ability to cluster in foci that are observed to occur either spontaneously or induced by DNA damage [4,13]. Foci distribution of C-terminal GFP tagged versions of Yen1 was compared (Fig 4 and S4 and S5 Tables; S6 Fig and S6 and S7 Tables). Mutating Yen1 SIMs lead to a roughly 3-fold decrease, from 10% to 3%, of the proportion of cells containing detectable Yen1-GFP foci (Fig 4B). This effect was more pronounced in a *mus81*Δ background, where 30% of cells contain spontaneous Yen1-GFP foci. In absence of Mus81, mutation in the SIMs of Yen1 lead approximately to a 10-fold decrease, from 30% to 3–4%, of Yen1-GFP foci (Fig 4B). These foci were equally decreased in the presence of exogenous damages induced by MMS in either a *MUS81* wild-type or null background, suggesting that Yen1 SIMs are equally important to properly localize the protein to spontaneous damaged sites and exogenous damaged sites (Fig 4B, and S4 and S5 Tables).

### The absence of Yen1’s SIMs prevents accumulation of the Yen1 fraction targeted by Slx5-Slx8

**Fig 5:**
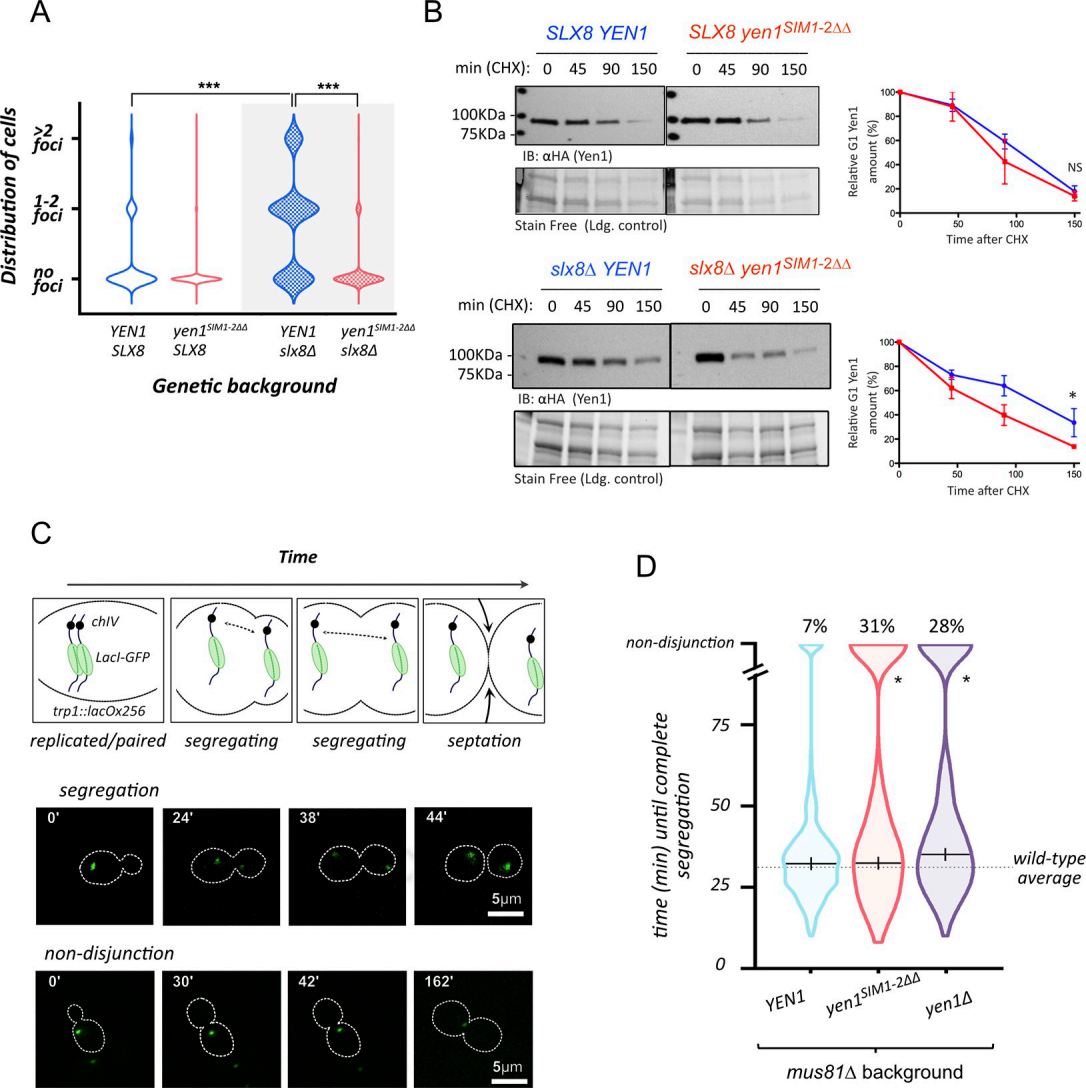
Mutation in the SIMs of Yen1 prevents foci accumulation in *slx8Δ* cells during G2/M and impacts chromosome segregation in a *mus81*Δ background. **(A)** Cells containing a deletion of *SLX8* were observed for their distribution of foci of the different variants of Yen1-GFP. Violin plots display the distribution of cells and asterisks denote statistical significance in a Chi-square test at P <0.0001 (***), see S8 Table. **(B)** Cells from the indicated genotypes were arrested in G1 and released in the presence of cycloheximide with samples being taken at the indicated time points. Total protein extracts were inspected by immunoblot for the presence of Yen1-HA and their intensity quantified relative to the loading control obtained by stain-free imaging of the gels (BioRad). Relative amounts of Yen1 are plotted in the graph to facilitate comparison (+/- SD). **(C)** Diagram showing chromosome segregation in cells harboring a lacO/GFP-LacI array tag on chromosome VII. To discriminate cells with timely chromosome segregation from those presenting aberrant segregation (delayed segregation or non-disjunction) a 2 hrs limit of observation was implemented. Two sets of representative actual images of a normal segregation pattern and a non-disjunction pattern are shown below the diagram. **(D)** Over 400 cells per strain were individually counted and are represented in violin plots according to the time spent to segregate the lacO/lacI array. The median segregation time is indicated excluding cells with non-disjunctions. Statistical relevance of the differences observed between the number of non-disjunctions of the different strains was determined by the Chi-square test at P<0.0001, see S9 Table.

We have demonstrated in a previous work a role of the Slx5-Slx8 SUMO-targeted ubiquitin ligase in the removal of a subset of Yen1 from the nucleus during the transition from G1 to S phase [13]. As a result, cells defective in *SLX8* show a persistence of Yen1 foci that take a longer time to disperse [13]. The accumulation of Yen1 foci in a *slx8*Δ strain is coupled to increased Yen1 SUMOylation, and occurs despite the presence of a functional Mus81-Mms4 nuclease [13]. A deletion of *SLX8* in the strain bearing the mutations in Yen1 SIMs did not increase the number of Yen1 foci that remained scarcely detectable (Fig 5A, S8 Table), indicating that Yen1 foci in an *slx8*Δ strain are SIM-dependent.

We have also demonstrated in our previous work that a wave of Yen1 turnover can be detected at the G1-S transition, and Yen1 levels are quickly decreased after a G1 synchronous release in the presence of the protein translation inhibitor cycloheximide [13]. In a *slx8*Δ strain, a fraction of the Yen1 protein pool remains un-degraded in such experiment, this fraction reflecting the protein pool requiring the specific SUMO-directed targeted degradation. Since the Yen1^SIM1-2ΔΔ^ mutant no longer forms foci and barely gets SUMOylated (Fig 2A), we anticipated that it would entirely be exposed to the degradation process that eliminates the bulk of non chromatin-bound Yen1 at the G1/S transition, independently of Slx5-Slx8 [13]. Accordingly, while the expected persistent Yen1 fraction was detected in the *slx8*Δ strain during a synchronous release from G1 under cycloheximide inhibition (Fig 5B), the double SIM mutant protein presented no persistent fraction, and the protein degradation happened at similar rates to that observed in *SLX8* wild-type backgrounds (Fig 5B).

### Impaired SUMO-directed localization induces an increase in untimely chromosome segregation

The presence of both *mus81*Δ and *yen1*Δ deletions makes cells synergistically sensitive to drugs like MMS, as stated before, and increases spontaneous chromosome mis-segregation events monitored either by dedicated genetic systems [5,48] or by a direct observation of fluorescent-tagged chromosomes during mitotic divisions [13] (Fig 5C). We compared the mitoses of single *mus81*Δ cells to those of cells carrying *mus81*Δ and the allele with the two mutated SIMs. Similar to what we could observe in a *mus81Δ yen1Δ* control strain, *mus81*Δ cells with the mutated Yen1 SIMs displayed an increased number of segregation issues.

As it can be observed in the violin plots displaying the time that individual-monitored cells spent to fully segregate the fluorescent tag, mutating both *YEN1* SIMs in a *mus81*Δ background does not impact the average time until complete chromosome segregation compared to *mus81*Δ cells producing WT Yen1 (Fig 5D). However, it led to a roughly four-fold increase (from 7% to 31%) of the proportion of cells that suffered chromosome segregation defects within the 2 hrs of video-microscopy observation (Fig 5D and S9 Table). The high number of chromosome mis-segregations detected for the Yen1 SIM mutant in a *mus81*Δ background is in line with that observed in cells completely lacking Yen1, clearly pointing to a faulty function of Yen1 when SIMs are impaired.

### Mutation in the SIMs of Yen1 reduces the formation of crossing over after a single DSB

**Fig 6:**
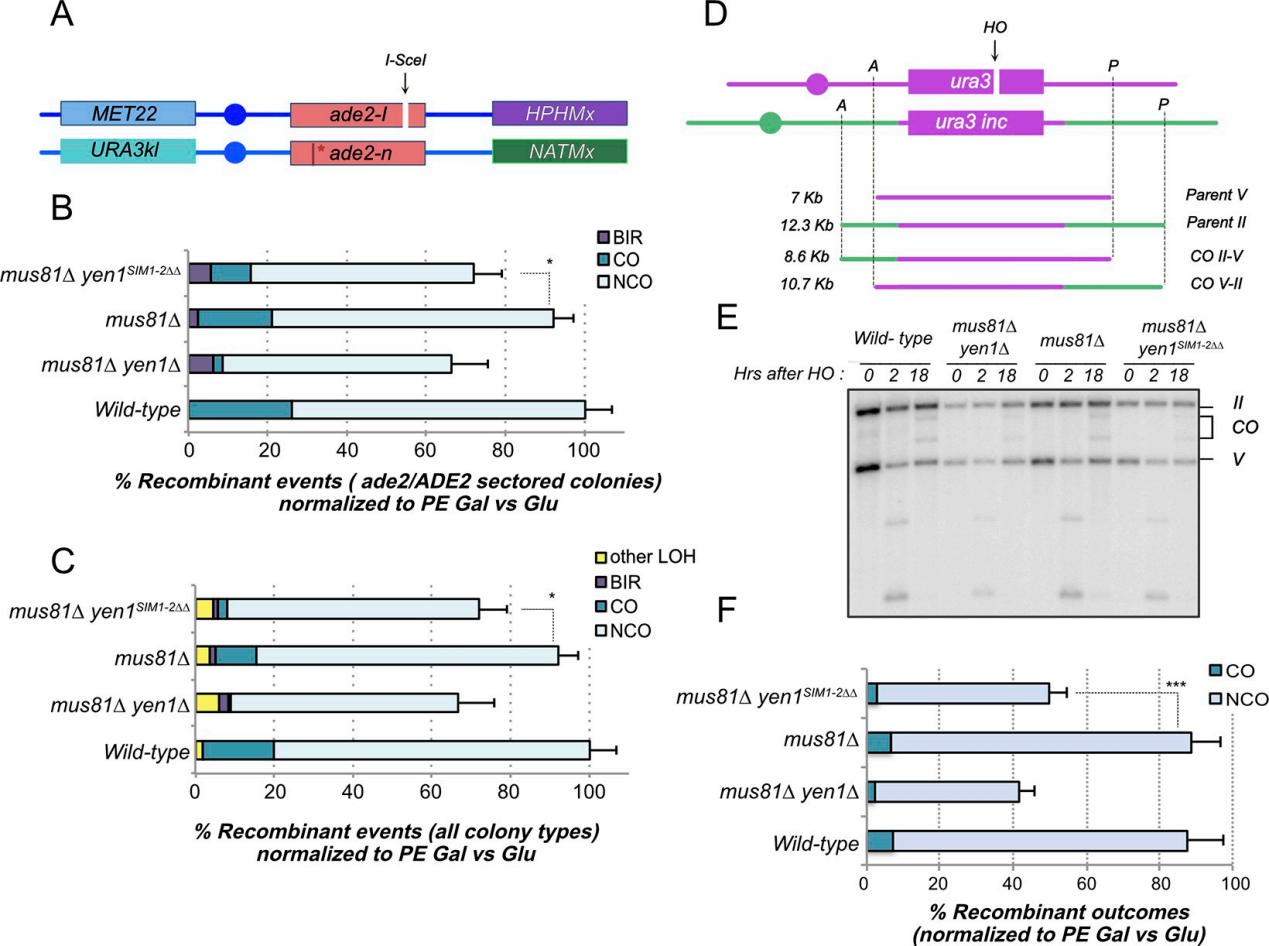
Crossover formation is impaired in cells containing the mutant version of Yen1 inactivating both SIMs in a *mus81Δ* background. **(A)** Diagram showing the chromosome XV based DSB-induced recombination reporter. **(B)** Recombination outcomes in red-white (ade2/ADE2) sectored colonies of the indicated strains, normalized to their Plating Efficiency (PE) in galactose compared to glucose. **(C)** Recombination outcomes combining the results obtained for all types of colonies (full red, full white and sectored) of the indicated strains, normalized to their Plating Efficiency (PE) in galactose compared to glucose. Statistical significance for B and C was determined by the Chi-square test at P<0.05, see S10 Table. **(D)** Diagram showing the chromosome II-V based ectopic DSB-induced recombination reporter and its expected outcomes during physical analysis. **(E)** Representative southern blot analysis of the indicated strains after genomic DNA digestion with the restriction enzymes as highlighted in diagram D and hybridization with a radiolabeled probe against the *URA3* locus. **(F)** Quantification of at least three independent southern blot analyses is plotted relative to PE (galactose vs glucose). Statistical significance was determined by the Student T-test at P<0.05, see S11 Table.

To further browse the implications of the presence of a defective SUMO-interacting Yen1 allele for the actual resolution of recombination intermediates, we decided to analyze the level of crossover (CO) formation in two widely used tests that estimate the CO levels after a single DSB formation [3,5,49] (Fig 6A and 6D). In accordance with the increased sensitivity to different DNA damaging agents observed for the Yen1 allele carrying the SIM mutations when combined with a *mus81Δ* background (Fig 2), we detected a decreased formation of crossovers in this genotype after the induction of a single DSB in a diploid tester strain [5], half-way to the phenotype observed with a double mutant carrying both *mus81*Δ and *yen1*Δ deletions (Fig 6B, 6C and S10 Table). The decrease in crossover formation was paralleled by an increase in break-induced replication (BIR) events (Fig 6B, 6C and S10 Table). Using an ectopic recombination assay [49] (Fig 6D), we detected a decrease in viability after the induction of an HO cut site in chromosome II in the *mus81*Δ *yen1*^*SIM1*-2ΔΔ^ strain, already signaling a defective crossover resolution resulting in a number of unviable events (Fig 6F). This survival decrease probably reflects a BIR increase that in this test leads to lethality caused by the loss of essential genes in the chromosome II distal arm. The number of crossovers quantified by southern blotting analysis of the survivors showed a nearly 50% reduction in the crossover yields in *mus81*Δ *yen1*^*SIM1*-2ΔΔ^ cells (Fig 6E, 6F and S11 Table), not significantly different from the levels detected in *mus81Δ yen1Δ* cells. Inactivation of the SIM1 motif alone achieved a similar reduction in CO yields and viability for this test, while the mutation introduced in the second motif alone did not have a significant effect (S7 Fig and S11 Table).

## Discussion

In the present work, we aimed to understand whether the Yen1 nuclease depends on interactions with SUMOylated partners to act accurately and promptly on its substrates. We have shown that besides being SUMOylated, Yen1 can also interact non-covalently with SUMOylated chains and SUMO monomers through at least two SUMO-interacting motifs in its C-terminal region (Fig 1). These SIMs mediate interaction with SUMO in either a classical yeast two-hybrid assay or pull-down assays (retention assays), with either immobilized GST-Smt3 or pre-polymerized poly-(6HIS)-Smt3 coupled to a Cobalt HisPur Superflow agarose matrix (Fig 1). Although our experiments point to a non-covalent interaction between Yen1 and SUMO in these experiments, we also have demonstrated that the absence of such interaction in a mutant variant of Yen1, modified in critical residues of its SIMs, leads to a nearly complete loss of direct SUMOylation of Yen1 *in vivo* when analyzed by denaturing pull-downs from cell extracts (Fig 3A). Our Yen1 SIM mutant acts thus as an *in vivo* SUMO-less variant without requiring a large number of Lysine substitutions, which can sometimes result in the protein’s destabilization. Previous reports suggested covalent SUMOylation occurring in the C-terminal region of Yen1 is responsible for the Smt3 interaction detected in a yeast two-hybrid assay [41]. Although our results are in general agreement with this observation, we conclude that it is non-covalent interaction mediated by the two SIMs that leads to a covalent modification of Yen1. This was confirmed by the SUMO-retention experiments and the 6His-Smt3 pull-down experiments, common approaches used to validate SIMs in other proteins showing SUMOylation [36,50–52]. While direct Yen1 SUMOylation depends strongly on the presence of the identified SIM motifs, we conclude that those are not necessary to mediate interaction with the SUMOylation machinery *per se*, as we can observe full SUMOylation patterns after an *in vitro* SUMOylation reaction (Fig 3B) and we also detect residual forms of SUMOylated Yen1 in our pull-down assays displaying the regular band pattern of *in vivo* SUMOylated Yen1 (Fig 3A), and not the absence of these forms that is obtained when Siz1 and Siz2 are removed [13]. However, we conclude that this direct SUMOylation of Yen1 is largely prevented in the cells by a faulty localization *via* SIMs to specific nuclear sites. Accordingly, while the absence of the SIMs in Yen1 has no effect on its catalytic activity (Fig 3C), we have detected a sub-optimal function of these mutants in the cells, leading to phenotypes of chromosome mis-segregation and DNA damage sensitivity similar to those observed for a null mutant in combination with a deletion of the partially redundant cell’s major resolvase activity mediated by the heterodimer Mus81-Mms4 (Figs 2 and 5) [5]. Indeed, these phenotypes correlate with the inability of the SIM mutant of Yen1 to properly localize in mitotic cells in previously characterized sub-nuclear localizations as foci, mainly at the vicinity of the nucleolus when cells are not exposed to exogenous DNA damage [13] (Fig 4). The impaired localization not only correlates with a sub-optimal function of Yen1 in response to spontaneous damages under normal growth conditions, but also decreases the number of crossing over that can be observed after single DSB induction in two different settings (Fig 6). While inactivation of both SIMs identified in the C-terminal region is necessary to impair non-covalent binding to SUMO, the mutation we introduced in SIM1 seems to achieve stronger phenotypes alone than the one in SIM2. We can speculate that SIM1 would thus be the main SIM in Yen1, with SIM2 playing a secondary role by further stabilizing interactions with SUMO that may imply a multi- or poly-SUMOylated partner.

Our results are in line with previous reports indicating a local enrichment of multiple SUMOylated proteins together with free Smt3 and the SUMOylation machinery during HR [35] that will also occur when persistent recombination intermediates are revealed during anaphase. While SUMOylation has been previously shown to play important roles in the fine-tuning of DNA repair processes, our study highlights the importance of SUMOylation for genome maintenance processes occurring in anaphase, and probably disconnected to previous SUMOylation cascades influencing HR proteins. Several key proteins acting in anaphase, like Condensin subunits and the chromosomal passenger complex (CPC) components, have been described to be SUMOylated [20,53,54]. It is thus of great interest to continue studying Yen1 functional interactions in conditions that are transient and ephemeral, and determine which other factors ensure prompt Yen1 recruitment to its activity sites during its anaphase activity window, thus influencing the delicate balance between chromosome segregation and genome integrity.

## Materials and methods

### Yeast strains and growth conditions

*S*. *cerevisiae* strains used in this study are derivatives of the W303c background and are listed in S1 Table. The Yen1-FX-GFP allele was made by inserting a Factor X site and the GFP epitope from pGAD-Yen1-GFP [55] between amino acids D753 and S754 at the C-terminus of Yen1 using dedicated oligonucleotides and was cloned into TOPO-pYES2 (Invitrogen) to allow controlled expression by galactose induction. All plasmid derivatives are listed in S2 Table. Mutants in the different designated loci where either obtained by crossing or by gene replacement with the indicated selective cassettes. Cells were typically grown in YP (1% yeast extract; 2% peptone) or SC media with alternatively 2% glucose, 2% raffinose or 2% galactose in strains under inducible conditions. A modified medium (SC with 0.17% YNB without ammonium sulfate, 0.1% proline and 0.003% SDS) was used for the Smt3 pull-down assays.

### Western blot analyses

If not stated otherwise, proteins were extracted by the TCA (trichloroacetic acid) method. For routine monitoring, samples were loaded into 7.5% Tris-glycine stain-free pre-casted gels (BioRad). Samples from pull-downs analyses were loaded into 3–8% gradient NuPAGE Tris-Acetate gels (ThermoFisher). Gels were transferred using a semi-dry transfer machine (BioRad) to PVDF membranes and hybridized with the appropriate antibodies in 5% w/v nonfat dry milk, 1X TBST buffer. Antibodies for anti-HA-HRP (3F10, Roche), anti-Smt3 (B. Palancade), anti-Pgk1-HRP (22C5D8, Abcam) were used at the suggested dilutions and revealed using an ECL reagent (Advansta). When required, HRP-conjugated secondary antibodies from Cell Signaling were used at 1/10000 dilution.

### Microscopy and cell biology methods

Live cell imaging was performed with a spinning disk confocal microscope (CSU-W1, Yokogawa), with an electron multiplying charge device camera (ANDOR Zyla sCMOS) and a ×60/1.35 numerical aperture objective at 30°C. Cells were centrifuged and plated as a droplet between an SC agarose pad and a glass slice [56]. Images were recorded with 17 z-sections with 0.5 μm spacing for each wavelength at a time. Video recordings were built with images taken every 2 minutes. Metamorph was used for image acquisition, and analysis was performed using Image J-Fiji [57].

For Yen1 foci observations, cells were grown in SC medium without uracil (SC-URA 2% raffinose), GFP-Yen1 was induced in a short burst of 30 min with galactose at 2%, followed by addition of glucose at 2%. For acute exposure to DNA damage, cells were treated with MMS 0.01% for 15 min at room temperature and were washed once with fresh SC-URA 2% glucose before continuing the experiment. Aliquots were taken at the indicated times. Cells showing an accumulation of spots were measured at maximum projection of the GFP channel. Statistical significance was determined by the χ2 test using contingency tables with the number of cells observed in each different category.

For segregation monitoring using strains with the lacO/GFP-LacI array, all cells were recorded for a duration of 2 hrs minimum in their agarose pads. Individual cells were identified with an ongoing chromosome segregation. To determine segregation duration, a start point was determined as the signature S-phase bud was the smallest yet discernable. At this point, only one foci of GFP-tagged chromosome fluorescent markers is visible. The cell is followed until the dot separates in two and stays durably in the daughter cells. The ending time point is the last frame of definitive separation of the fluorescent foci. The duration of the movement of the two separate dots was reported for each individual cell under monitoring, cells with dots moving together for the whole duration of the time-lapse were classified as non-disjunction and their segregation time was not used to establish the average segregation time.

### SUMOylation assays and Smt3-bound retention assay

In *ex vivo* SUMOylation assays, the wild-type or mutant Yen1-HA was produced from a pYES2 vector and immuno-precipitated from cell lysates as described [13]. Eluates were subjected to SUMO conjugation and ligation as described [58].

For Smt3-retention assays, 6x-His-Smt3 was purified from BL21 *E*.*coli* cells using a Ni-NTA affinity column (Qiagen) following manufacturer indications. Smt3 protein was eluted with 250 mM imidazole before being dialyzed using a G2 Slide-a-Lyzer cassette (Thermo Fisher) with a 10 kDa cut-off. Purified Smt3 was subjected to a self-conjugation reaction by adding Aos1-Uba2 and Ubc9 and ATP as described [58] and the reaction was subjected to a second purification in a Cobalt HisPur Superflow agarose matrix (Thermo Fisher) to generate poly-Smt3 retention column. Equal amounts of Yen1 or its mutant were added to the non eluted matrix containing poly-Smt3 bound in E buffer (20 mM NaH_2_PO_4_, 300 mM NaCl, 5 mM Imidazole, pH 7.4) and binding was allowed for 60 min at 4°C. Mini-columns were then centrifuged to remove the buffer and non-retained proteins, washed 5 times in washing buffer (E buffer 12.5 mM Imidazole) and eluted in denaturing conditions with Laemmli buffer at 95°C. The eluates were loaded into 4–15% SDS-PAGE gradient gels and immunoblotted. GST-Smt3 retention assays were performed as described [36] with purified GST-Smt3 obtained by expression of pGEX-4T1-Smt3 into BL21 *E*.*coli* cells. Briefly, purified GST-Smt3 was incubated with glutathione matrix during 40 min at 4°C in GST buffer (25 mM Tris-HCl (pH8.0), 150mM NaCl, 1mM DTT). Then matrix was washed before to load purified wild-type or mutated Yen1. Mixtures were incubated 3 min at 4°C before glutathione matrix washing. The retained proteins were analyzed by immuno-blot analysis.

### Cycloheximide chase experiments

Cycloheximide chase experiments were essentially done as reported [13]. Cultures grown in SC complete modified media (0.1% proline 0.017% YNB without ammonium sulfate, 0.0003% SDS) were diluted to OD_600_ = 0.2 and synchronized with alpha factor (3 μM) for 2 h. Once synchronized, cells were treated with cycloheximide (250 μg/ml) in fresh media, to inhibit proteins new synthesis, and released from the G1 arrest. Samples were taken at indicated time points and analyzed by TCA extraction and western blotting.

### Denaturing histidine pull-downs

For 6xHIS-Smt3 pull-downs, strains containing the expression vectors or the control empty plasmid were grown in SC-LEU modified medium (0.1% proline, 0.017% YNB without ammonium sulfate). Cells were allowed to grow to OD_600_ = 0.3 when CuSO_4_ was added at 100 μM final concentration in a volume of 100 ml. After 1 h, MMS was added to 0.3% and cells were collected 3 hrs later. Cells where lysed under denaturing conditions and SUMO-conjugated proteins where isolated and analyzed by western blot using a Nu-PAGE Tris-acetate 3–8% gradient gel, basically as previously described [13]. In *dna2-2* strains (LEU2), a plasmid expressing under galactose induction Flag-6His-Smt3 (URA3) was used instead of the Cu-inducible.

### Synthetic DNA substrates and Yen1 resolvase activity assays

The synthetic HJ-X0 was prepared by annealing the Cy5-X0-1, X0-2, X0-3 and X0-4 oligonucleotides (Sigma-Aldrich) in a buffer containing 50 mM Tris-HCl (pH 7.5), 10 mM MgCl_2_, 100 mM NaCl as described [47]. The annealing product was analyzed in a native PAGE to verify the presence of a HJ structure. To test Yen1 activity, an enzymatic reaction was performed in 10 μl cleavage buffer (50 mM Tris-HCl (pH 7.5), 1 mM MgCl_2_, 1 mM DTT) containing 25 nM of Cy5-labled HJ X0 substrate, and equal amounts of immunoprecipitated Yen1 or its SIM-defective mutant. After incubation at 30°C for 1 h, the reaction was stopped by adding 2.5 μl of stop buffer (100 mM Tris-HCl (7.5), 50 mM EDTA, 2.5% SDS, 10 mg/ml proteinase K) and further incubated for 30 min at 37°C. Cleavage products were migrated in 10% native PAGE, scanned using a Typhoon FLA 9500 Biomolecular Imager and the images were analyzed with ImageQuant (GE Healthcare).

### DSB-induced recombination assays

The diploid recombination assays were performed as described previously (for a detailed protocol see [48]). The reporter diploid strains that contain 2 *ade2* hetero-alleles were cleaved by induction of I-*Sce*I in its *ade2*-I allele and allowed to repair with its ade2-n allele under non-selective conditions to give rise to either ADE2 or *ade2*-n repair products in three types of colonies (red, white and sectored). We scored each recombinant colony with its two recombination events (for each repaired sister chromatid) and considering the possible segregation patterns in daughter cells. Frequencies of the recombination events were normalized to the galactose vs glucose plating efficiency. The distribution of CO and NCO in the ectopic recombination assay based in chromosomes V and II [49] were addressed by southern blot hybridization of *Apa*LI-*Pvu*II digested genomic DNA from cell populations growing in YP-raffinose after galactose induction of HO. Membranes were hybridized with a *URA3* radiolabeled probe and results were normalized relative to the galactose versus glucose plating efficiency of the strains as described [3].

## Supporting information

S1 Fig Homology and conservation of the Yen1 SUMO interacting motifs. **(A)** Alignment of Yen1’s SIM1 (Aa 635–646) to already characterized SIMs in Slx5, Elg1 and Rad18 presenting an aliphatic core flanked by acidic (D/E), phosphorylatable (S/T) or polar residues (R/K). **(B)** Alignment of putative SIM motifs found in Yen1 sequences from different yeast species matching the architecture of Yen1 SIM1 in *S*. *cerevisiae* **(C)** Disposition of SIM1 in *S*. *cerevisiae* and other yeast species relative to the SIM2 and the conserved domains of the bi-partite NLS (containing a regulatory CDK1 site).
(EPS)

S2 Fig Phosphorylation status and cellular distribution of Yen1 SIM variants. **(A)** Comparison of the phosphorylation status of the different Yen1 variants with mutated SIMs during the cell cycle. Cells were released from an alpha factor synchronization (G1) and samples were monitored at the indicated times. Total protein extracts were separated in a phos-tag containing PAGE. **(B)** Representative cells of the indicated cell cycle phases carrying the indicated GFP tagged Yen1 variants. A Histone (Hta2) mark with mCherry is also shown to delineate the nuclear compartment. The dotted line defines the boundaries of cells as observed in the DIC images.
(EPS)

S3 Fig Effect of alternative sets of mutations on SIM1. **(A)** Diagram depicting the two sets of mutations used to modify SIM1. **(B)** Sensitivity of cells carrying the indicated genotypes to different DNA damaging agents was tested by spotting serial dilutions in media with the indicated doses. Plates were grown for 3 days at 30°C. **(C)** Analysis of denaturing histidine PD against 6His-Smt3 of the indicated strains treated with 0.3% MMS.
(EPS)

S4 Fig Genetic interactions of *DNA2* alleles and *YEN1* alleles. **(A)** Analysis of tetrad dissection from meiosis of the indicated diploids. Unviable genetic combinations are depicted as dotted red triangles. Legend of genotypes is presented at the right of the images. (**B**) Independent clones from different dissected tetrads were tested for sensitivity to the DNA damaging agents by spotting serial dilutions of cultures in media with the indicated doses. An heterogeneity of sensitivities was detected for cells containing *dna2-2* alleles, suggesting the accumulation of suppressors during propagation of these clones. (**C)** Sensitivity of cells carrying the indicated genotypes to different DNA damaging agents was tested by spotting serial dilutions in media with the indicated doses. Plates in A/B/C were grown for 3 days at 30°C. **(D)** Analysis of denaturing histidine PD of 6His-Smt3 of the indicated untreated strains.
(EPS)

S5 Fig Quantification of Yen1 products after *in vitro* SUMOylation. **(A**) Adjusted intensity of the upper forms and the lower unmodified form of Yen1 was calculated for blots of the *in vitro* SUMO ligation reactions (containing Siz2) to determine the relative abundance of SUMOylated products (%) (**B**) Average of the quantification of relative SUMOylation products from two independent ligation assays plotted as a function of the time of reaction (+/-SD).
(EPS)

S6 Fig Yen1 foci distribution in individual and combined SIM mutants. Strains bearing an internal -HA tag version of Yen1 with the indicated mutation and a GFP- tagged copy in an inducible plasmid were monitored for Yen1 foci. Violin plots show the distribution of G2/M cells according to the presence of 1–2 foci, >2 foci or no foci at all. Between 100 and 400 cells were counted from 2–3 independent trials depending on the constructs. Left: Results obtained in a *MUS81* wild-type background. Right: Results obtained in a *mus81*Δ background. Statistics and raw numbers can be found in S6 and S7 Tables.
(EPS)

S7 Fig Crossover levels after DSB induction in Yen1 SIM mutants. **(A)** Southern blot analysis of the indicated strains treated as highlighted in Fig 6 and hybridized with a probe at the URA3 locus. **(B)** Quantification of at least three independent southern blot analyses is plotted relative to PE (galactose vs glucose). Statistical significance was determined by the Student T-test at P<0.05, see S11 Table.
(EPS)

S1 Table Yeast strains used in this study. (PDF)

S2 Table Plasmids used in this study. (PDF)

S3 Table P values of the statistical analysis (Student’s T-Test) of the differences of survival as displayed in Fig 2. (PDF)

S4 Table Number of cells distributed in the different categories of Yen1-GFP foci for the indicated genetic backgrounds as displayed in Fig 4B violin plots. (PDF)

S5 Table Chi-square statistical results of the analysis of the categories of cells (according to its Yen1-GFP foci, Fig 4B). (PDF)

S6 Table Number of cells distributed in the different categories for Yen1-GFP foci as displayed in S6 Fig violin plots (no MMS treatment). (PDF)

S7 Table Chi-square statistical results of the analysis of the categories of cells (according to its Yen1-GFP foci, S6 Fig). (PDF)

S8 Table Number of cells distributed in the different categories for Yen1-GFP foci in an *slx8*Δ background, as displayed in Fig 5 violin plots. (PDF)

S9 Table Distribution of cells in the chromosome segregation experiments of Fig 5. (PDF)

S10 Table V Raw numbers obtained from the analysis of the colonies in the experiments with the diploid crossover reporter (Fig 6). (PDF)

S11 Table Values for quantification of crossovers using an ectopic recombination reporter strain and statistic significance (Figs 6 and S7). (PDF)

## References

[1] Mitchel K, Zhang H, Welz-Voegele C, Jinks-Robertson S. Molecular structures of crossover and noncrossover intermediates during gap repair in yeast: implications for recombination. Mol Cell. 2010;38(2):211–22. doi: 10.1016/j.molcel.2010.02.028 ; PubMed Central PMCID: PMC2865147. 20417600 PMC2865147

[2] Symington LS, Rothstein R, Lisby M. Mechanisms and regulation of mitotic recombination in Saccharomyces cerevisiae. Genetics. 2014;198(3):795–835. doi: 10.1534/genetics.114.166140 ; PubMed Central PMCID: PMC4224172. 25381364 PMC4224172

[3] Mazon G, Symington LS. Mph1 and Mus81-Mms4 prevent aberrant processing of mitotic recombination intermediates. Mol Cell. 2013;52(1):63–74. doi: 10.1016/j.molcel.2013.09.007 ; PubMed Central PMCID: PMC3818723. 24119400 PMC3818723

[4] Garcia-Luis J, Machin F. Mus81-Mms4 and Yen1 resolve a novel anaphase bridge formed by noncanonical Holliday junctions. Nat Commun. 2014;5:5652. doi: 10.1038/ncomms6652 . 25466415

[5] Ho CK, Mazon G, Lam AF, Symington LS. Mus81 and Yen1 promote reciprocal exchange during mitotic recombination to maintain genome integrity in budding yeast. Mol Cell. 2010;40(6):988–1000. doi: 10.1016/j.molcel.2010.11.016 ; PubMed Central PMCID: PMC3021384. 21172663 PMC3021384

[6] Matos J, Blanco MG, Maslen S, Skehel JM, West SC. Regulatory control of the resolution of DNA recombination intermediates during meiosis and mitosis. Cell. 2011;147(1):158–72. doi: 10.1016/j.cell.2011.08.032 ; PubMed Central PMCID: PMC3560330. 21962513 PMC3560330

[7] Gallo-Fernandez M, Saugar I, Ortiz-Bazan MA, Vazquez MV, Tercero JA. Cell cycle-dependent regulation of the nuclease activity of Mus81-Eme1/Mms4. Nucleic Acids Res. 2012;40(17):8325–35. doi: 10.1093/nar/gks599 ; PubMed Central PMCID: PMC3458551. 22730299 PMC3458551

[8] Szakal B, Branzei D. Premature Cdk1/Cdc5/Mus81 pathway activation induces aberrant replication and deleterious crossover. EMBO J. 2013;32(8):1155–67. doi: 10.1038/emboj.2013.67 ; PubMed Central PMCID: PMC3630363. 23531881 PMC3630363

[9] Gritenaite D, Princz LN, Szakal B, Bantele SC, Wendeler L, Schilbach S, et al. A cell cycle-regulated Slx4-Dpb11 complex promotes the resolution of DNA repair intermediates linked to stalled replication. Genes Dev. 2014;28(14):1604–19. doi: 10.1101/gad.240515.114 ; PubMed Central PMCID: PMC4102767. 25030699 PMC4102767

[10] Bastin-Shanower SA, Fricke WM, Mullen JR, Brill SJ. The mechanism of Mus81-Mms4 cleavage site selection distinguishes it from the homologous endonuclease Rad1-Rad10. Mol Cell Biol. 2003;23(10):3487–96. doi: 10.1128/MCB.23.10.3487-3496.2003 ; PubMed Central PMCID: PMC164751. 12724407 PMC164751

[11] Blanco MG, Matos J, West SC. Dual control of Yen1 nuclease activity and cellular localization by Cdk and Cdc14 prevents genome instability. Mol Cell. 2014;54(1):94–106. doi: 10.1016/j.molcel.2014.02.011 ; PubMed Central PMCID: PMC3988869. 24631285 PMC3988869

[12] Eissler CL, Mazon G, Powers BL, Savinov SN, Symington LS, Hall MC. The Cdk/cDc14 module controls activation of the Yen1 holliday junction resolvase to promote genome stability. Mol Cell. 2014;54(1):80–93. doi: 10.1016/j.molcel.2014.02.012 ; PubMed Central PMCID: PMC3988236. 24631283 PMC3988236

[13] Talhaoui I, Bernal M, Mullen JR, Dorison H, Palancade B, Brill SJ, et al. Slx5-Slx8 ubiquitin ligase targets active pools of the Yen1 nuclease to limit crossover formation. Nat Commun. 2018;9(1):5016. Epub 2018/11/28. doi: 10.1038/s41467-018-07364-x ; PubMed Central PMCID: PMC6258734. 30479332 PMC6258734

[14] Johnson ES. Protein modification by SUMO. Annu Rev Biochem. 2004;73:355–82. Epub 2004/06/11. doi: 10.1146/annurev.biochem.73.011303.074118 . 15189146

[15] Flotho A, Melchior F. Sumoylation: a regulatory protein modification in health and disease. Annu Rev Biochem. 2013;82:357–85. Epub 2013/06/12. doi: 10.1146/annurev-biochem-061909-093311 . 23746258

[16] Nie M, Boddy MN. Cooperativity of the SUMO and Ubiquitin Pathways in Genome Stability. Biomolecules. 2016;6(1):14. doi: 10.3390/biom6010014 ; PubMed Central PMCID: PMC4808808. 26927199 PMC4808808

[17] Psakhye I, Jentsch S. Protein group modification and synergy in the SUMO pathway as exemplified in DNA repair. Cell. 2012;151(4):807–20. Epub 2012/11/06. doi: 10.1016/j.cell.2012.10.021 . 23122649

[18] Sarangi P, Zhao X. SUMO-mediated regulation of DNA damage repair and responses. Trends Biochem Sci. 2015;40(4):233–42. Epub 2015/03/18. doi: 10.1016/j.tibs.2015.02.006 ; PubMed Central PMCID: PMC4380773. 25778614 PMC4380773

[19] Johnson ES, Gupta AA. An E3-like factor that promotes SUMO conjugation to the yeast septins. Cell. 2001;106(6):735–44. Epub 2001/09/27. doi: 10.1016/s0092-8674(01)00491-3 . 11572779

[20] Takahashi Y, Kahyo T, Toh EA, Yasuda H, Kikuchi Y. Yeast Ull1/Siz1 is a novel SUMO1/Smt3 ligase for septin components and functions as an adaptor between conjugating enzyme and substrates. J Biol Chem. 2001;276(52):48973–7. Epub 2001/09/29. doi: 10.1074/jbc.M109295200 . 11577116

[21] Zhao X, Blobel G. A SUMO ligase is part of a nuclear multiprotein complex that affects DNA repair and chromosomal organization. Proc Natl Acad Sci U S A. 2005;102(13):4777–82. Epub 2005/03/02. doi: 10.1073/pnas.0500537102 ; PubMed Central PMCID: PMC555716. 15738391 PMC555716

[22] Sacher M, Pfander B, Hoege C, Jentsch S. Control of Rad52 recombination activity by double-strand break-induced SUMO modification. Nat Cell Biol. 2006;8(11):1284–90. Epub 2006/10/03. doi: 10.1038/ncb1488 . 17013376

[23] Pfander B, Moldovan GL, Sacher M, Hoege C, Jentsch S. SUMO-modified PCNA recruits Srs2 to prevent recombination during S phase. Nature. 2005;436(7049):428–33. Epub 2005/06/03. doi: 10.1038/nature03665 . 15931174

[24] Hoege C, Pfander B, Moldovan GL, Pyrowolakis G, Jentsch S. RAD6-dependent DNA repair is linked to modification of PCNA by ubiquitin and SUMO. Nature. 2002;419(6903):135–41. Epub 2002/09/13. doi: 10.1038/nature00991 . 12226657

[25] Cremona CA, Sarangi P, Yang Y, Hang LE, Rahman S, Zhao X. Extensive DNA damage-induced sumoylation contributes to replication and repair and acts in addition to the mec1 checkpoint. Mol Cell. 2012;45(3):422–32. Epub 2012/01/31. doi: 10.1016/j.molcel.2011.11.028 ; PubMed Central PMCID: PMC3340930. 22285753 PMC3340930

[26] Bonner JN, Choi K, Xue X, Torres NP, Szakal B, Wei L, et al. Smc5/6 Mediated Sumoylation of the Sgs1-Top3-Rmi1 Complex Promotes Removal of Recombination Intermediates. Cell Rep. 2016;16(2):368–78. Epub 2016/07/05. doi: 10.1016/j.celrep.2016.06.015 ; PubMed Central PMCID: PMC5051638. 27373152 PMC5051638

[27] Beauclair G, Bridier-Nahmias A, Zagury JF, Saib A, Zamborlini A. JASSA: a comprehensive tool for prediction of SUMOylation sites and SIMs. Bioinformatics. 2015;31(21):3483–91. Epub 2015/07/05. doi: 10.1093/bioinformatics/btv403 . 26142185

[28] Kerscher O. SUMO junction-what’s your function? New insights through SUMO-interacting motifs. EMBO Rep. 2007;8(6):550–5. Epub 2007/06/05. doi: 10.1038/sj.embor.7400980 ; PubMed Central PMCID: PMC2002525. 17545995 PMC2002525

[29] Minty A, Dumont X, Kaghad M, Caput D. Covalent modification of p73alpha by SUMO-1. Two-hybrid screening with p73 identifies novel SUMO-1-interacting proteins and a SUMO-1 interaction motif. J Biol Chem. 2000;275(46):36316–23. Epub 2000/08/30. doi: 10.1074/jbc.M004293200 . 10961991

[30] Song J, Zhang Z, Hu W, Chen Y. Small ubiquitin-like modifier (SUMO) recognition of a SUMO binding motif: a reversal of the bound orientation. J Biol Chem. 2005;280(48):40122–9. Epub 2005/10/06. doi: 10.1074/jbc.M507059200 . 16204249

[31] Song J, Durrin LK, Wilkinson TA, Krontiris TG, Chen Y. Identification of a SUMO-binding motif that recognizes SUMO-modified proteins. Proc Natl Acad Sci U S A. 2004;101(40):14373–8. Epub 2004/09/25. doi: 10.1073/pnas.0403498101 ; PubMed Central PMCID: PMC521952. 15388847 PMC521952

[32] Hofmann K, Falquet L. A ubiquitin-interacting motif conserved in components of the proteasomal and lysosomal protein degradation systems. Trends Biochem Sci. 2001;26(6):347–50. Epub 2001/06/19. doi: 10.1016/s0968-0004(01)01835-7 . 11406394

[33] Fisher RD, Wang B, Alam SL, Higginson DS, Robinson H, Sundquist WI, et al. Structure and ubiquitin binding of the ubiquitin-interacting motif. J Biol Chem. 2003;278(31):28976–84. Epub 2003/05/17. doi: 10.1074/jbc.M302596200 . 12750381

[34] Gareau JR, Lima CD. The SUMO pathway: emerging mechanisms that shape specificity, conjugation and recognition. Nat Rev Mol Cell Biol. 2010;11(12):861–71. Epub 2010/11/26. doi: 10.1038/nrm3011 ; PubMed Central PMCID: PMC3079294. 21102611 PMC3079294

[35] Jentsch S, Psakhye I. Control of nuclear activities by substrate-selective and protein-group SUMOylation. Annu Rev Genet. 2013;47:167–86. Epub 2013/09/11. doi: 10.1146/annurev-genet-111212-133453 . 24016193

[36] Parker JL, Ulrich HD. A SUMO-interacting motif activates budding yeast ubiquitin ligase Rad18 towards SUMO-modified PCNA. Nucleic Acids Res. 2012;40(22):11380–8. Epub 2012/10/05. doi: 10.1093/nar/gks892 ; PubMed Central PMCID: PMC3526273. 23034805 PMC3526273

[37] Bermudez-Lopez M, Villoria MT, Esteras M, Jarmuz A, Torres-Rosell J, Clemente-Blanco A, et al. Sgs1’s roles in DNA end resection, HJ dissolution, and crossover suppression require a two-step SUMO regulation dependent on Smc5/6. Genes Dev. 2016;30(11):1339–56. Epub 2016/06/15. doi: 10.1101/gad.278275.116 ; PubMed Central PMCID: PMC4911932. 27298337 PMC4911932

[38] Cappadocia L, Mascle XH, Bourdeau V, Tremblay-Belzile S, Chaker-Margot M, Lussier-Price M, et al. Structural and functional characterization of the phosphorylation-dependent interaction between PML and SUMO1. Structure. 2015;23(1):126–38. Epub 2014/12/17. doi: 10.1016/j.str.2014.10.015 . 25497731

[39] Cook CE, Hochstrasser M, Kerscher O. The SUMO-targeted ubiquitin ligase subunit Slx5 resides in nuclear foci and at sites of DNA breaks. Cell Cycle. 2009;8(7):1080–9. Epub 2009/03/10. doi: 10.4161/cc.8.7.8123 ; PubMed Central PMCID: PMC2700622. 19270524 PMC2700622

[40] Parnas O, Zipin-Roitman A, Pfander B, Liefshitz B, Mazor Y, Ben-Aroya S, et al. Elg1, an alternative subunit of the RFC clamp loader, preferentially interacts with SUMOylated PCNA. EMBO J. 2010;29(15):2611–22. Epub 2010/06/24. doi: 10.1038/emboj.2010.128 ; PubMed Central PMCID: PMC2928695. 20571511 PMC2928695

[41] Bauer SL, Chen J, Astrom SU. Helicase/SUMO-targeted ubiquitin ligase Uls1 interacts with the Holliday junction resolvase Yen1. PLoS One. 2019;14(3):e0214102. Epub 2019/03/22. doi: 10.1371/journal.pone.0214102 ; PubMed Central PMCID: PMC6428284. 30897139 PMC6428284

[42] Budd ME, Tong AH, Polaczek P, Peng X, Boone C, Campbell JL. A network of multi-tasking proteins at the DNA replication fork preserves genome stability. PLoS Genet. 2005;1(6):e61. Epub 2005/12/06. doi: 10.1371/journal.pgen.0010061 ; PubMed Central PMCID: PMC1298934. 16327883 PMC1298934

[43] Olmezer G, Levikova M, Klein D, Falquet B, Fontana GA, Cejka P, et al. Replication intermediates that escape Dna2 activity are processed by Holliday junction resolvase Yen1. Nat Commun. 2016;7:13157. doi: 10.1038/ncomms13157 ; PubMed Central PMCID: PMC5093310. 27779184 PMC5093310

[44] Falquet B, Olmezer G, Enkner F, Klein D, Challa K, Appanah R, et al. Disease-associated DNA2 nuclease-helicase protects cells from lethal chromosome under-replication. Nucleic Acids Res. 2020;48(13):7265–78. Epub 2020/06/17. doi: 10.1093/nar/gkaa524 ; PubMed Central PMCID: PMC7367196. 32544229 PMC7367196

[45] Budd ME, Reis CC, Smith S, Myung K, Campbell JL. Evidence suggesting that Pif1 helicase functions in DNA replication with the Dna2 helicase/nuclease and DNA polymerase delta. Mol Cell Biol. 2006;26(7):2490–500. doi: 10.1128/MCB.26.7.2490-2500.2006 ; PubMed Central PMCID: PMC1430326. 16537895 PMC1430326

[46] Ip SC, Rass U, Blanco MG, Flynn HR, Skehel JM, West SC. Identification of Holliday junction resolvases from humans and yeast. Nature. 2008;456(7220):357–61. doi: 10.1038/nature07470 . 19020614

[47] Benson FE, West SC. Substrate specificity of the Escherichia coli RuvC protein. Resolution of three- and four-stranded recombination intermediates. J Biol Chem. 1994;269(7):5195–201. Epub 1994/02/18. . 8106501

[48] Mazon G, Lam AF, Ho CK, Kupiec M, Symington LS. The Rad1-Rad10 nuclease promotes chromosome translocations between dispersed repeats. Nat Struct Mol Biol. 2012;19(9):964–71. doi: 10.1038/nsmb.2359 ; PubMed Central PMCID: PMC3443319. 22885325 PMC3443319

[49] Agmon N, Yovel M, Harari Y, Liefshitz B, Kupiec M. The role of Holliday junction resolvases in the repair of spontaneous and induced DNA damage. Nucleic Acids Res. 2011;39(16):7009–19. doi: 10.1093/nar/gkr277 ; PubMed Central PMCID: PMC3167605. 21609961 PMC3167605

[50] Hecker CM, Rabiller M, Haglund K, Bayer P, Dikic I. Specification of SUMO1- and SUMO2-interacting motifs. J Biol Chem. 2006;281(23):16117–27. Epub 2006/03/10. doi: 10.1074/jbc.M512757200 . 16524884

[51] Xie Y, Kerscher O, Kroetz MB, McConchie HF, Sung P, Hochstrasser M. The yeast Hex3.Slx8 heterodimer is a ubiquitin ligase stimulated by substrate sumoylation. J Biol Chem. 2007;282(47):34176–84. Epub 2007/09/13. doi: 10.1074/jbc.M706025200 . 17848550

[52] Balakirev MY, Mullally JE, Favier A, Assard N, Sulpice E, Lindsey DF, et al. Wss1 metalloprotease partners with Cdc48/Doa1 in processing genotoxic SUMO conjugates. Elife. 2015;4. Epub 2015/09/09. doi: 10.7554/eLife.06763 ; PubMed Central PMCID: PMC4559962. 26349035 PMC4559962

[53] D’Amours D, Stegmeier F, Amon A. Cdc14 and condensin control the dissolution of cohesin-independent chromosome linkages at repeated DNA. Cell. 2004;117(4):455–69. Epub 2004/05/13. doi: 10.1016/s0092-8674(04)00413-1 . 15137939

[54] Dasso M. Emerging roles of the SUMO pathway in mitosis. Cell Div. 2008;3:5. Epub 2008/01/26. doi: 10.1186/1747-1028-3-5 ; PubMed Central PMCID: PMC2265688. 18218095 PMC2265688

[55] Kosugi S, Hasebe M, Tomita M, Yanagawa H. Systematic identification of cell cycle-dependent yeast nucleocytoplasmic shuttling proteins by prediction of composite motifs. Proc Natl Acad Sci U S A. 2009;106(25):10171–6. doi: 10.1073/pnas.0900604106 ; PubMed Central PMCID: PMC2695404. 19520826 PMC2695404

[56] Tran PT, Paoletti A, Chang F. Imaging green fluorescent protein fusions in living fission yeast cells. Methods. 2004;33(3):220–5. Epub 2004/05/26. doi: 10.1016/j.ymeth.2003.11.017 . 15157889

[57] Schindelin J, Arganda-Carreras I, Frise E, Kaynig V, Longair M, Pietzsch T, et al. Fiji: an open-source platform for biological-image analysis. Nat Methods. 2012;9(7):676–82. doi: 10.1038/nmeth.2019 ; PubMed Central PMCID: PMC3855844. 22743772 PMC3855844

[58] Bretes H, Rouviere JO, Leger T, Oeffinger M, Devaux F, Doye V, et al. Sumoylation of the THO complex regulates the biogenesis of a subset of mRNPs. Nucleic Acids Res. 2014;42(8):5043–58. Epub 2014/02/07. doi: 10.1093/nar/gku124 ; PubMed Central PMCID: PMC4005672. 24500206 PMC4005672

