# Supplementary material for: SUMO-mediated recruitment allows timely function of the Yen1 nuclease in mitotic cells
Source: PLoS Genet. 2022 Mar 25;18(3):e1009860. doi: 10.1371/journal.pgen.1009860 (PMC8986097; doi:10.1371/journal.pgen.1009860)
Supplement: S1 Table — (PDF) [file pgen.1009860.s008.pdf]

S1 Table. Yeast strains used in this study.

**Strain**	**Genotype ***	**Source or reference**
**Strains for general purpose**		
GM84	*MATa ADE2 yen1Δ::klURA3 ura3Δ::HphMX*	[1]
GM93-5D	*MATα ADE2 yen1Δ::klURA3 mus81Δ::KanMX*	[1]
GM98-4B	*MATa ADE2 YEN1-HA ura3Δ::HphMX*	[1]
GM98-5A	*MATa ADE2 YEN1-HA ura3-1*	[1]
GM98-7B	*MATα ADE2 YEN1-HA mus81Δ::KanMX ura3Δ::HphMX*	[1]
GM98-6A	*MATα ADE2 YEN1-HA mus81Δ::KanMX ura3-1*	[1]
GM481-2A	*MATa ADE2 YEN1-HA mus81Δ::KanMX ura3Δ::HphMX*	[1]
GM395-5C	*MATa ADE2 yen1::HIS3 ura3-1*	[1]
GM395-14C	*MATa ADE2 yen1::HIS3 mus81Δ::KanMX ura3-1*	[1]
GM411-6B	*MATa ADE2 yen1*$^{-SIM1\Delta}$*-HA ura3-1*	This study
GM592-2B	*MATa ADE2 yen1*$^{-SIM2\Delta}$*-HA ura3-1*	This study
GM719-16C	*MATa ADE2 yen1*$^{-SIM1\text{-}2\Delta\Delta}$*-HA ura3-1*	This study
GM411-1D	*MATa ADE2 yen1*$^{-SIM1\Delta}$*-HA mus81Δ::KanMX ura3-1*	This study
GM592-1C	*MATa ADE2 yen1*$^{-SIM2\Delta}$*-HA mus81Δ::KanMX ura3-1*	This study
GM719-2A	*MATa ADE2 yen1*$^{-SIM1\text{-}2\Delta\Delta}$*-HA mus81Δ::KanMX ura3-1*	This study
GM481-3D	*MATa ADE2 YEN1-HA slx8Δ::KanMX ura3Δ::HphMX*	This study
GM898-7A	*MATa ADE2 yen1*$^{-SIM1\text{-}2\Delta\Delta}$*-HA slx8Δ::KanMX ura3Δ::HphMX*	This study
GM975	*MATa ade2-1 dna2-2::LEU2*	J. Campbell Lab
GM979-3A	*MATa ADE2 dna2-2::LEU2 YEN1-HA ura3Δ::HphMX*	This study
GM979-2C	*MATα ADE2 dna2-2::LEU2 YEN1-HA ura3Δ::HphMX*	This study
GM980-4C	*MATa ADE2 dna2-2::LEU2 yen1*$^{-SIM1\text{-}2\Delta\Delta}$*-HA ura3Δ::HphMX*	This study
GM980-4D	*MATα ADE2 dna2-2::LEU2 yen1*$^{-SIM1\text{-}2\Delta\Delta}$*-HA ura3-1*	This study
GM973	*MATα ADE2 dna2Δ::KANMX YEN1-HA pif1Δ*	This study
GM974	*MATα ADE2 dna2Δ::KANMX yen1*$^{-SIM1\text{-}2\Delta\Delta}$*-HA pif1Δ*	This study
GM1015-1B	*MATa ADE2 yen1*$^{-SIM1\psi}$ *-HA*	This study
GM1015-3B	*MATa ADE2 yen1*$^{-SIM1\psi}$ *-HA mus81Δ::KanMX*	This study

**Strains for cell biology**		
GM120-12A	MATα ADE2 yen1::HIS3 mus81Δ::KanMX his3-11:pCUP1-GFP12-LacI12:HIS3 trp1-1:256LacO:TRP1 ura3Δ::HphMX	[1]
GM304-6A	MATα ADE2 Yen1-HA mus81::KanMX his3-11:pCUP1-GFP12-LacI12:HIS3 trp1-1:256LacO:TRP1 ura3Δ::HphMX	[1]
GM747-6D	MATα ADE2 Yen1$^{-SIM1-2\Delta\Delta}$-HA mus81::KanMX his3-11:pCUP1-GFP12-LacI12:HIS3 trp1-1:256LacO:TRP1 ura3Δ::HphMX	This study
GM361-3C	MATa ADE2 YEN1-HA HTA1-mCherry::HphMX	This study
GM726-15C	MATa ADE2 YEN1-HA mus81Δ::KanMX HTA1-mCherry::HphMX	This study
GM427-3D	MATa ADE2 yen1$^{-SIM1\Delta}$-HA mus81Δ::KanMX HTA1-mCherry::HphMX	This stufy
GM687-3A	MATa ADE2 yen1$^{-SIM2\Delta}$-HA HTA1-mCherry::HphMX	This study
GM687-6B	MATa ADE2 yen1$^{-SIM2\Delta}$-HA mus81Δ::KanMX HTA1-mCherry::HphMX	This study
GM720-12C	MATa ADE2 yen1$^{-SIM1-2\Delta\Delta}$-HA HTA1-mCherry::HphMX	This study
GM720-2D	MATα ADE2 yen1$^{-SIM1-2\Delta\Delta}$-HA mus81Δ::KanMX HTA1-mCherry::HphMX	This study
**Strains for crossover monitoring**		
LSY2205-24D	MATα ade2-I lys2::GAL-ISCEI his3::HphMX4 yen1::HIS3	[2]
LSY2202-42A	MAT**a** ade2-n his3::NatMX4 met22::klURA3 yen1::HIS3	[2]
LSY2205-77B	MAT**a** ade2-I lys2::GAL-ISCEI his3::HphMX4 mus81::KanMX6 yen1::HIS3	[2]
LSY2202-19D	MATα ade2-n his3::NatMX4 met22::klURA3 mus81::KanMX6 yen1::HIS3	[2]
GM379-4C	MATa YEN1-HA ade2-I lys2Δ::pGal-ISceI his3Δ::HphMX	[1]
GM379-13C	MATα YEN1-HA mus81Δ::KanMX ade2-I lys2::GAL-ISCEI his3::HphMX4	[1]
GM387-22B	MATα YEN1-HA ade2-n his3::NatMX4 met22::klURA3	[1]
GM387-5A	MAT**a** YEN1-HA mus81Δ::KanMX ade2-n his3::NatMX4 met22::klURA3	[1]
GM802-12D	MATα yen1$^{-SIM1-2\Delta\Delta}$-HA ade2-I lys2::GAL-ISCEI his3::HphMX4	This study
GM801-4A	MAT**a** yen1$^{-SIM1-2\Delta\Delta}$-HA ade2-n his3::NatMX4 met22::klURA3	This study
GM802-6B	MATa yen1$^{-SIM1-2\Delta\Delta}$-HA mus81Δ::KanMX ade2-I lys2::GAL-ISCEI his3::HphMX4	This study
GM801-46D	MATα yen1$^{-SIM1-2\Delta\Delta}$-HA mus81Δ::KanMX ade2-n his3::NatMX4 met22::klURA3	This study
GM401-2D	MATa-inc ADE2 YEN1-HA mus81Δ::KanMX ura3::HOcs (V) lys2::ura3-Hocs inc (5.6Kb) (II) ade3::pGAL-HO	This study
GM401-11C	MATa-inc ADE2 YEN1-HA ura3::HOcs (V) lys2::ura3-HOcs inc (5.6Kb) (II) ade3::pGAL-HO	[1]
GM999-5A	MATa-inc ADE2 yen1$^{-SIM1\Delta}$-HA mus81Δ::KanMX ura3::HOcs (V) lys2::ura3-HOcs inc (5.6Kb) (II) ade3::pGAL-HO	This study

GM994-5D	*MATa-inc ADE2 yen1$^{-SIM2\Delta}$-HA mus81Δ::KanMX ura3::HOcs (V) lys2::ura3-HOcs inc (5.6Kb) (II) ade3::pGAL-HO*	This study
GM880-6D	*MATa-inc ADE2 yen1$^{-SIM1-2\Delta\Delta}$-HA mus81Δ::KanMX ura3::HOcs (V) lys2::ura3-HOcs inc (5.6Kb) (II) ade3::pGAL-HO*	This study
LSY2548-29C	*MATa-inc ADE2 yen1Δ mus81Δ::KanMX ura3::HOcs (V) lys2::ura3-HOcs inc (5.6Kb) (II) ade3::pGAL-HO*	[3]
**Strains for Two-Hybrid**		
PJ69-4a	*MAT**a** trp1-901leu2-3.12 ura3-52 his3-200 gal4Δ gal80Δ LYS2::GAL1-HIS3 GAL2-ADE2 met2::GAL7-lacz*	Stan Fields Lab [4]
Pj69-4alpha	*MATα trp1-901leu2-3.12 ura3-52 his3-200 gal4Δ gal80Δ LYS2::GAL1-HIS3 GAL2-ADE2 met2::GAL7-lacz*	Stan Fields Lab [4]

*If not stated otherwise strains background is the W303 genotype (his3-11, 15 leu2-3, 112 trp1-1 ade2-1 can1-100), only mating type and differences from the standard genotype are listed. Specific strains in other backgrounds are defined. Yen1$^{SIM1\Delta}$ summarizes mutations D635A D636A D637A in Yen1, Yen1$^{SIM2\Delta}$ represents V675A E677A mutations. Yen1$^{SIM1\Psi}$ summarizes mutations I640A F641A V642A in Yen1.

References

1. Talhaoui I, Bernal M, Mullen JR, Dorison H, Palancade B, Brill SJ, et al. Slx5-Slx8 ubiquitin ligase targets active pools of the Yen1 nuclease to limit crossover formation. Nat Commun. 2018;9(1):5016. Epub 2018/11/28. doi: 10.1038/s41467-018-07364-x. PubMed PMID: 30479332; PubMed Central PMCID: PMCPMC6258734.
2. Ho CK, Mazon G, Lam AF, Symington LS. Mus81 and Yen1 promote reciprocal exchange during mitotic recombination to maintain genome integrity in budding yeast. Mol Cell. 2010;40(6):988-1000. doi: 10.1016/j.molcel.2010.11.016. PubMed PMID: 21172663; PubMed Central PMCID: PMCPMC3021384.
3. Mazon G, Lam AF, Ho CK, Kupiec M, Symington LS. The Rad1-Rad10 nuclease promotes chromosome translocations between dispersed repeats. Nat Struct Mol Biol. 2012;19(9):964-71. doi: 10.1038/nsmb.2359. PubMed PMID: 22885325; PubMed Central PMCID: PMCPMC3443319.
4. James P, Halladay J, Craig EA. Genomic libraries and a host strain designed for highly efficient two-hybrid selection in yeast. Genetics. 1996;144(4):1425-36. PubMed PMID: 8978031; PubMed Central PMCID: PMCPMC1207695.
