# Supplementary material for: SUMO-mediated recruitment allows timely function of the Yen1 nuclease in mitotic cells
Source: PLoS Genet. 2022 Mar 25;18(3):e1009860. doi: 10.1371/journal.pgen.1009860 (PMC8986097; doi:10.1371/journal.pgen.1009860)
Supplement: S2 Table — (PDF) [file pgen.1009860.s009.pdf]

S2 Table. Plasmids used in this study.

Plasmid	Description
**pYES2**	*pYES2 (URA3) empty vector, GAL inducible*
**pYES2-Yen1-HA**	*pYES2 (URA3) derivative, GAL inducible*
**pYES2-yen1$^{SIM1\Delta}$-HA**	*pYES2 (URA3) derivative, GAL inducible*
**pYES2-yen1$^{SIM2\Delta}$-HA**	*pYES2 (URA3) derivative, GAL inducible*
**pYES2-yen1$^{SIM1\text{-}2\Delta\Delta}$-HA**	*pYES2-TOPO (URA3) derivative, GAL inducible*
**pYES2-yen1$^{SIM1\psi}$-HA**	*pYES2-TOPO (URA3) derivative, GAL inducible*
**pYES2-Yen1-GFP**	*pYES2-TOPO (URA3) derivative, GAL inducible*
**pYES2- yen1$^{SIM1\Delta}$-GFP**	*pYES2-TOPO (URA3) derivative, GAL inducible*
**pYES2- yen1$^{SIM2\Delta}$-GFP**	*pYES2-TOPO (URA3) derivative, GAL inducible*
**pYES2- yen1$^{SIM1\text{-}2\Delta\Delta}$-GFP**	*pYES2-TOPO (URA3) derivative, GAL inducible*
**pYES2-GFP-Yen1**	*pYES2-TOPO (URA3) derivative, GAL inducible*
**pYES2-GFP-yen1$^{SIM1\Delta}$**	*pYES2-TOPO (URA3) derivative, GAL inducible*
**pYES2-GFP-yen1$^{SIM2\Delta}$**	*pYES2-TOPO (URA3) derivative, GAL inducible*
**pYES2-GFP-yen1$^{SIM1\text{-}2\Delta\Delta}$**	*pYES2-TOPO (URA3) derivative, GAL inducible*
**pRS315**	*empty vector (LEU2)*
**p1346**	*Cu inducible (LEU2) encoding 6xHIS-Smt3 (from B. Palancade)*
**p1067 His-Flag-Smt3 (LEU2)**	Gal inducible (URA3) encoding His-Flag-Smt3 *(from B. Palancade)*
**pOAD**	*AD fusion empty vector (TRP1) (from Stan Fields)*
**pOAD-Smt3**	*AD fusion with Smt3 (TRP1), derived from pOAD (from Stan Fields)*
**pDBD-Yen1**	*DBD fusion with Yen1 (LEU2), derived from pOBD2 (from Stan Fields)*
**pDBD-ΔYen1(354-759)**	*DBD fusion with a truncated Yen1 (LEU2), derived from pOBD2 (from Stan Fields)*
**pDBD-Yen1$^{SIM1\Delta}$**	*DBD fusion with Yen1-SIM1Δ (LEU2), derived from pOBD2 (from Stan Fields)*
**pDBD-Yen1$^{SIM2\Delta}$**	*DBD fusion with Yen1-SIM2Δ (LEU2), derived from pOBD2 (from Stan Fields)*
**pDBD-Yen1$^{SIM1\text{-}2\Delta\Delta}$**	*DBD fusion with Yen1-SIM1-2ΔΔ (LEU2), derived from pOBD2 (from Stan Fields)*
**pGEX-4T2**	*Vector expressing GST*
**pGEX-4T2-Smt3**	*Vector expressing a GST fusion with Smt3 in bacteria (AmpR), derived from pGEX-4T2*
**pET21b-6His-Smt3**	*Vector expressing a 6xHis fusion with Smt3 in bacteria (AmpR), derived from pET21b*
**pET21b-6His-Smt3-K$^{11\text{-}15\text{-}19}$R**	*Vector expressing a 6His fusion with Smt-KR mutant in bacteria (AmpR), derived from pET21b*
