# Supplementary material for: SUMO-mediated recruitment allows timely function of the Yen1 nuclease in mitotic cells
Source: PLoS Genet. 2022 Mar 25;18(3):e1009860. doi: 10.1371/journal.pgen.1009860 (PMC8986097; doi:10.1371/journal.pgen.1009860)
Supplement: S3 Table — (PDF) [file pgen.1009860.s010.pdf]

S3 Table. P values from the statistical analysis (Student's T-Test) of the differences of survival displayed in Figure 2.

**Dose**	***P values of T-Test compared to mus81Δ YEN1***			
	vs mus81Δ yen1^{SIM1Δ}	*vs mus81Δ yen1^{SIM2Δ}*	*vs mus81Δ yen1$^{SIM1-2ΔΔ}$*	*vs mus81Δ yen1Δ*
0.00375% MMS	0.039	NS	0.0017	--
0.0025% MMS	0.024	NS	0.0042	0.0016
4 μg/ml Zeocin	NS	NS	0.0026	0.0002
20 mM HU	0.057	NS	0.02	0.0093
40 mM HU	0.017	NS	0.0057	--
