# Supplementary material for: SUMO-mediated recruitment allows timely function of the Yen1 nuclease in mitotic cells
Source: PLoS Genet. 2022 Mar 25;18(3):e1009860. doi: 10.1371/journal.pgen.1009860 (PMC8986097; doi:10.1371/journal.pgen.1009860)
Supplement: S4 Table — (PDF) [file pgen.1009860.s011.pdf]

S4 Table. Number of cells distributed in the different categories for Yen1-GFP foci with a *MUS81* genetic background as displayed in Figure 4B violin plots.

		Cell categories (Yen1-GFP foci)		
	**Total Cells**	**No Foci**	**1-2 Foci**	**>2 Foci**
MUS81 background				
**No MMS**				
MUS81 YEN1	514	416	81	17
MUS81 *yen1*$^{SIM1\text{-}2\Delta\Delta}$	582	566	15	1
**1h30 after MMS**				
MUS81 YEN1	152	43	35	74
MUS81 *yen1*$^{SIM1\text{-}2\Delta\Delta}$	178	136	27	15
**3h30 after MMS**				
MUS81 YEN1	203	120	63	20
MUS81 *yen1*$^{SIM1\text{-}2\Delta\Delta}$	201	191	10	0
mus81Δ background				
**No MMS**				
mus81Δ YEN1	453	239	165	49
mus81Δ yen1$^{SIM1\text{-}2\Delta\Delta}$	422	390	32	0
**1h30 after MMS**				
mus81Δ YEN1	330	34	137	159
mus81Δ yen1$^{SIM1\text{-}2\Delta\Delta}$	487	243	183	61
**3h30 after MMS**				
mus81Δ YEN1	301	90	124	87
mus81Δ yen1$^{SIM1\text{-}2\Delta\Delta}$	263	209	49	5
