# Supplementary material for: SUMO-mediated recruitment allows timely function of the Yen1 nuclease in mitotic cells
Source: PLoS Genet. 2022 Mar 25;18(3):e1009860. doi: 10.1371/journal.pgen.1009860 (PMC8986097; doi:10.1371/journal.pgen.1009860)
Supplement: S5 Table — (PDF) [file pgen.1009860.s012.pdf]

S5 Table. Chi-square statistical results of the analysis of the categories of cells (according to its Yen1 foci, Figure 4B).

MUS81 yen1$^{SIM1\text{-}2\Delta\Delta}$	Chi2 against *YEN1 MUS81* (for each condition)	
No MMS	**X2 (2, N = 1096) = 78,593 p < 0,00001**	***
1h30 after MMS	**X2 (2, N = 330) = 86,9543 p < 0,00001**	***
3h30 after MMS	**X2 (2, N = 404) = 73,8914 p < 0,00001**	***

mus81Δ yen1$^{SIM1\text{-}2\Delta\Delta}$	Chi2 against *YEN1 mus81Δ* (for each condition)	
No MMS	**X2 (2, N = 876) = 171,295 p < 0,00001**	***
1h30 after MMS	**X2 (2, N = 817) = 184,607 p < 0,00001**	***
3h30 after MMS	**X2 (2, N = 564) = 151,088 p < 0,00001**	***
