# Supplementary material for: SUMO-mediated recruitment allows timely function of the Yen1 nuclease in mitotic cells
Source: PLoS Genet. 2022 Mar 25;18(3):e1009860. doi: 10.1371/journal.pgen.1009860 (PMC8986097; doi:10.1371/journal.pgen.1009860)
Supplement: S6 Table — (PDF) [file pgen.1009860.s013.pdf]

S6 Table. Number of cells distributed in the different categories for Yen1-GFP foci as displayed in S6 Figure violin plots (no MMS treatment).

		Cell categories (Yen1-GFP foci)		
	**Total Cells**	**No Foci**	**1-2 Foci**	**>2 Foci**
MUS81 YEN1	514	416	81	17
MUS81 yen1^{SIM1Δ}	430	412	18	0
MUS81 yen1^{SIM2Δ}	430	379	43	8
MUS81 yen1$^{SIM1\text{-}2ΔΔ}$	582	566	15	1
mus81Δ YEN1	453	239	165	49
mus81Δ yen1^{SIM1Δ}	317	294	23	0
mus81Δ yen1^{SIM2Δ}	239	165	62	12
mus81Δ yen1$^{SIM1\text{-}2ΔΔ}$	422	390	32	0
