# Supplementary material for: SUMO-mediated recruitment allows timely function of the Yen1 nuclease in mitotic cells
Source: PLoS Genet. 2022 Mar 25;18(3):e1009860. doi: 10.1371/journal.pgen.1009860 (PMC8986097; doi:10.1371/journal.pgen.1009860)
Supplement: S7 Table — (PDF) [file pgen.1009860.s014.pdf]

S7 Table. Chi-square statistical results of the analysis of the categories of cells (according to its Yen1-GFP foci, S6 Figure).

	Chi2 against YEN1 MUS81	
MUS81 yen1$^{SIM1\Delta}$	**X2 (2, N = 945) = 47,4082, p < 0,00001**	***
MUS81 yen1$^{SIM2\Delta}$	**X2 (2, N = 944) = 9,2055, p = 0,010024**	**
MUS81 yen1$^{SIM1\text{-}2\Delta\Delta}$	**X2 (2, N = 1096) = 78,5932 p < 0,00001**	***
	Chi2 against *YEN1 mus81Δ*	
mus81Δ yen1$^{SIM1\Delta}$	**X2 (2, N = 771) = 139,6543 p < 0,00001**	***
mus81Δ yen1$^{SIM2\Delta}$	**X2 (2, N = 692) = 18,3041 p = 0,000106**	***
mus81Δ yen1$^{SIM1\text{-}2\Delta\Delta}$	**X2 (2, N = 876) = 171,295 p < 0,00001**	***
