# Supplementary material for: SUMO-mediated recruitment allows timely function of the Yen1 nuclease in mitotic cells
Source: PLoS Genet. 2022 Mar 25;18(3):e1009860. doi: 10.1371/journal.pgen.1009860 (PMC8986097; doi:10.1371/journal.pgen.1009860)
Supplement: S8 Table — (PDF) [file pgen.1009860.s015.pdf]

**S8 Table.** Number of cells distributed in the different categories for Yen1-GFP foci in a *slx8*Δ background, as displayed in Figure 5 violin plots.

| | Total Cells | Cell categories (Yen1-GFP foci) | | |
|---|---|---|---|---|
| | | No Foci | 1-2 Foci | >2 Foci |
| *slx8Δ YEN1* | 291 | 109 | 135 | 47 |
| *slx8Δ yen1*$^{SIM1\text{-}2\Delta\Delta}$ | 291 | 266 | 23 | 2 |

Chi-square analysis: **X2 (2, N = 582) = 186,4496 p < 0,00001**
