# Supplementary material for: SUMO-mediated recruitment allows timely function of the Yen1 nuclease in mitotic cells
Source: PLoS Genet. 2022 Mar 25;18(3):e1009860. doi: 10.1371/journal.pgen.1009860 (PMC8986097; doi:10.1371/journal.pgen.1009860)
Supplement: S9 Table — (PDF) [file pgen.1009860.s016.pdf]

S9 Table. Distribution of cells in the chromosome segregation experiments of Figure 5.

		Segregation categories	
	**Total Cells***	**Division completed****	**Non-disjunction**
mus81Δ YEN1	446	416	30
mus81Δ $yen1^{SIM1\text{-}2\Delta\Delta}$	412	284	128
mus81Δ yen1Δ	411	295	116

* The different trials are pooled for analysis. ** Cells that completed segregation are displayed pooled regardless of its segregation time.

Chi^2 *mus81Δ* $yen1^{SIM1\text{-}2\Delta}$ against *YEN1 mus81Δ* / **X2 (1, N = 858) = 84,4615 p < 0,00001**

Chi^2 *mus81Δ yen1Δ* against *YEN1 mus81Δ* / **X2 (1, N = 857) = 69,9369 p < 0,00001**
