# Supplementary material for: SUMO-mediated recruitment allows timely function of the Yen1 nuclease in mitotic cells
Source: PLoS Genet. 2022 Mar 25;18(3):e1009860. doi: 10.1371/journal.pgen.1009860 (PMC8986097; doi:10.1371/journal.pgen.1009860)
Supplement: S10 Table — (PDF) [file pgen.1009860.s017.pdf]

S10 Table. Raw numbers obtained from the analysis of the colonies in the experiments with the diploid crossover reporter (Figure 6).

Recombination types (sectored colonies)	*mus81Δ YEN1*	*mus81Δ yen1Δ*	*mus81Δ yen1$^{SIM1\text{-}2ΔΔ}$*
**BIR** (colonies/events)	6 / 6	15 / 15	16 / 16
**CO** (colonies/events)	21 / 42	3 / 6	14 / 28
**NCO** (colonies/events)	78 / 162	82 / 160	71 / 158

**Freeman-Halton Fisher's vs *mus81Δ***		<0.0001	0.027
**Chi-square vs *mus81Δ***		X^2 (2, 370)=26.3 P< 0.0001	X^2 (2, 412)=7.24 P= 0.027

Recombination types (full red colonies)	*mus81Δ YEN1*	*mus81Δ yen1Δ*	*mus81Δ yen1$^{SIM1\text{-}2ΔΔ}$*
**BIR** (colonies/events)	4 / 4	35 / 35	12 / 12
**CO** (colonies/events)	15 / 30	5 / 10	17 / 34
**NCO** (colonies/events)	118 / 240	378 / 791	334 / 680
**unassigned LOH** (colonies/events)	20 / 20	19 / 119	87 / 87

Recombination types (full white colonies)	*mus81Δ YEN1*	*mus81Δ yen1Δ*	*mus81Δ yen1$^{SIM1\text{-}2ΔΔ}$*
**BIR** (colonies/events)	2 / 2	2 / 2	1 / 1
**CO** (colonies/events)	5 / 10	1 / 2	2 / 4
**NCO** (colonies/events)	93 /188	211 / 424	362 / 725
**unassigned LOH** (colonies/events)	7 / 7	24 / 24	31 / 31

% Colony Types (ADE2)	*mus81Δ YEN1*	*mus81Δ yen1Δ*	*mus81Δ yen1$^{SIM1\text{-}2ΔΔ}$*
**Red (*ade2/ade2*)**	42.54 %	63.02 %	47.51 %
**Sectored (*ade2/ADE2*)**	28.45 %	9.33 %	10.66 %
**White (*ADE2/ADE2*)**	28.99 %	27.64 %	41.81 %
**Long Tract Conversion**	56.8 %	67.7 %	52.85 %
**Short Tract Conversion**	43.2 %	32.3 %	47.15 %
