# Supplementary material for: SUMO-mediated recruitment allows timely function of the Yen1 nuclease in mitotic cells
Source: PLoS Genet. 2022 Mar 25;18(3):e1009860. doi: 10.1371/journal.pgen.1009860 (PMC8986097; doi:10.1371/journal.pgen.1009860)
Supplement: S11 Table — (PDF) [file pgen.1009860.s018.pdf]

S11 Table. Values for quantification of crossovers using the ectopic recombination strain and statistic significances (Figures 6 and S7).

	MUS81 YEN1	*mus81Δ YEN1*	*mus81Δ yen1Δ*	*mus81Δ* $yen1^{SIM1\text{-}2\Delta\Delta}$	*mus81Δ* $yen1^{SIM1\Delta}$	*mus81Δ* $yen1^{SIM2\Delta}$
**P.E. Gal/Glu**<br>**SEM (#trials)**	87.7 %<br>9.9 (2)	88.8 %<br>3.9 (4)	41.9 %<br>7.9 (5)	49.9 %<br>4.7 (5)	54.4 %<br>8.8 (3)	78.8%<br>6.3 (2)
**Raw CO values (%)**	8.1/ 8.2/ 7.8/ 9.4	9.7/ 8.6/ 6.9/ 7.1/ 7.9/ 7.1/ 6.2/ 7.0	4.9 /4.7 / 5.6 / 5.9 / 5.2 / 5.5 / 3.9	6.0/ 6.0 /7.1/ 6.7/ 5.7/ 5.3/ 4.6	4.2 / 6.7 / 5.9	7.6 / 7.6 / 7.5
**Average Raw CO value (±SD)**	8.37% (±0.71)	7.57% (±1.11)	5.11% (±0.66)	5.91% (±0.81)	5.64% (±1.27)	7.59% (±0.02)
**Relative CO value (±SD)**	7.34% (±0.62)	6.71% (±0.99)	2.13% (±0.28)	3.00% (±0.41)	3.07% (±0.69)	5.97% (±0.02)
**Relative NCO value**	80.36 %	82.08 %	39.77 %	46.98 %	51.39 %	72.73 %
**T-Test PE vs *mus81Δ* (P value)**		___	0.01	0.0004	0.0429	___
**T-Test rel. CO vs *mus81Δ* (P value)**		___	0.0002	0.006	0.001	___
